# Evolution of cyclizing 5-aminolevulinate synthases in the biosynthesis of actinomycete secondary metabolites: outcomes for genetic screening techniques

**DOI:** 10.3389/fmicb.2015.00814

**Published:** 2015-08-05

**Authors:** Kateřina Petříčková, Alica Chroňáková, Tomáš Zelenka, Tomáš Chrudimský, Stanislav Pospíšil, Miroslav Petříček, Václav Krištůfek

**Affiliations:** ^1^Institute of Microbiology, Czech Academy of Sciences, v. v. i.Prague, Czech Republic; ^2^Institute of Soil Biology, Biology Centre, Czech Academy of Sciences, v. v. i.České Budějovice, Czech Republic

**Keywords:** 5-aminolevulinate synthase, C_5_N unit, *Streptomyces*, secondary metabolites, gene evolution, genetic screening, horizontal gene transfer

## Abstract

A combined approach, comprising PCR screening and genome mining, was used to unravel the diversity and phylogeny of genes encoding 5-aminolevulinic acid synthases (ALASs, *hemA* gene products) in streptomycetes-related strains. In actinomycetes, these genes were believed to be directly connected with the production of secondary metabolites carrying the C_5_N unit, 2-amino-3-hydroxycyclopent-2-enone, with biological activities making them attractive for future use in medicine and agriculture. Unlike “classical” primary metabolism ALAS, the C_5_N unit-forming cyclizing ALAS (cALAS) catalyses intramolecular cyclization of nascent 5-aminolevulinate. Specific amino acid sequence changes can be traced by comparison of “classical” ALASs against cALASs. PCR screening revealed 226 *hemA* gene-carrying strains from 1,500 tested, with 87% putatively encoding cALAS. Phylogenetic analysis of the *hemA* homologs revealed strain clustering according to putative type of metabolic product, which could be used to select producers of specific C_5_N compound classes. Supporting information was acquired through analysis of actinomycete genomic sequence data available in GenBank and further genetic or metabolic characterization of selected strains. Comparison of 16S rRNA taxonomic identification and BOX-PCR profiles provided evidence for numerous horizontal gene transfers of biosynthetic genes or gene clusters within actinomycete populations and even from non-actinomycete organisms. Our results underline the importance of environmental and evolutionary data in the design of efficient techniques for identification of novel producers.

## Introduction

5-aminolevulinate (ALA) is a key precursor of a huge family of essential tetrapyrrole compounds. Two distinct ALA biosynthetic pathways have evolved; that for archea, majority of bacteria, and plants where ALA is derived from glutamate via the C5 pathway. Some α-Proteobacteria, fungi, and animals form ALA through condensation of glycine and succinyl-CoA by *hemA*-encoded aminolevulinate synthase in so called C4 or Shemin pathway. Presence of both pathways in a single cell is extremely rare and their products are always temporally, spatially or functionally separated (Weinstein and Beale, 1983; Mayer and Beale, 1992; Yang and Hoober, 1995). We recently reported one example in actinomycetes, where the C5 pathway is devoted to primary metabolism and the C4 strictly toward feeding biosynthesis of secondary metabolites containing the ALA-derived cyclic moiety, the C_5_N unit, 2-amino-3-hydroxycyclopent-2-enone (Petříček et al., 2006; Supplementary Figure S1). These secondary metabolites have a range of structures and functions, including linear or aromatic polyketides, glycolipids, macrolides, peptide compounds, and others (Supplementary Figure S2). Many have pharmaceutically attractive biological activities and in some cases, the relevant biosynthetic gene clusters have already been cloned and characterized.

Recently, it has been shown that formation of the C_5_N unit and its attachment to core structures of antibiotics is directed by three enzymes: cyclizing 5-aminolevulinate synthase (cALAS encoded by a *hemA* homolog), amide synthase (AMS) and aminolevulinate-CoA ligase (ALL). These always appear to be encoded by three adjacent genes located within a relevant antibiotic biosynthetic gene cluster. We consider the presence of this gene triad to be a specific genetic marker of the C_5_N unit biosynthetic pathway that “tags” producers of C_5_N-containing metabolites among others. The key enzyme, cALAS, catalyzes not only condensation of glycine and succinyl-CoA, but also the subsequent cyclization of nascent ALA (Zhang et al., 2010, Supplementary Figure S1). The other two enzymes activate and attach the unit to the metabolite core.

The principal aim of our work was to show the potential of the *hemA*-targeted genetic screening in identification of novel putative biosynthetic gene clusters encoding C_5_N-containing metabolites. For the purpose, occurrence of *hemA* was mapped in a large diverse collection of natural streptomycete isolates and also in the collection of genomic data of actinomycetes in the GenBank. The next aim was to verify the hypothesis, that the specialized, cyclizing type of ALAS, and perhaps the entire gene triad responsible for the C_5_N biosynthesis, have evolved within individual biosynthetic gene clusters, which in many cases differ diametrically. *HemA* gene phylogeny should then reflect the diversity of synthesized chemical structures and should provide an important genetic clue in classification of novel putative producers.

## Materials and Methods

### Genome Scanning

**FIGURE 1:**
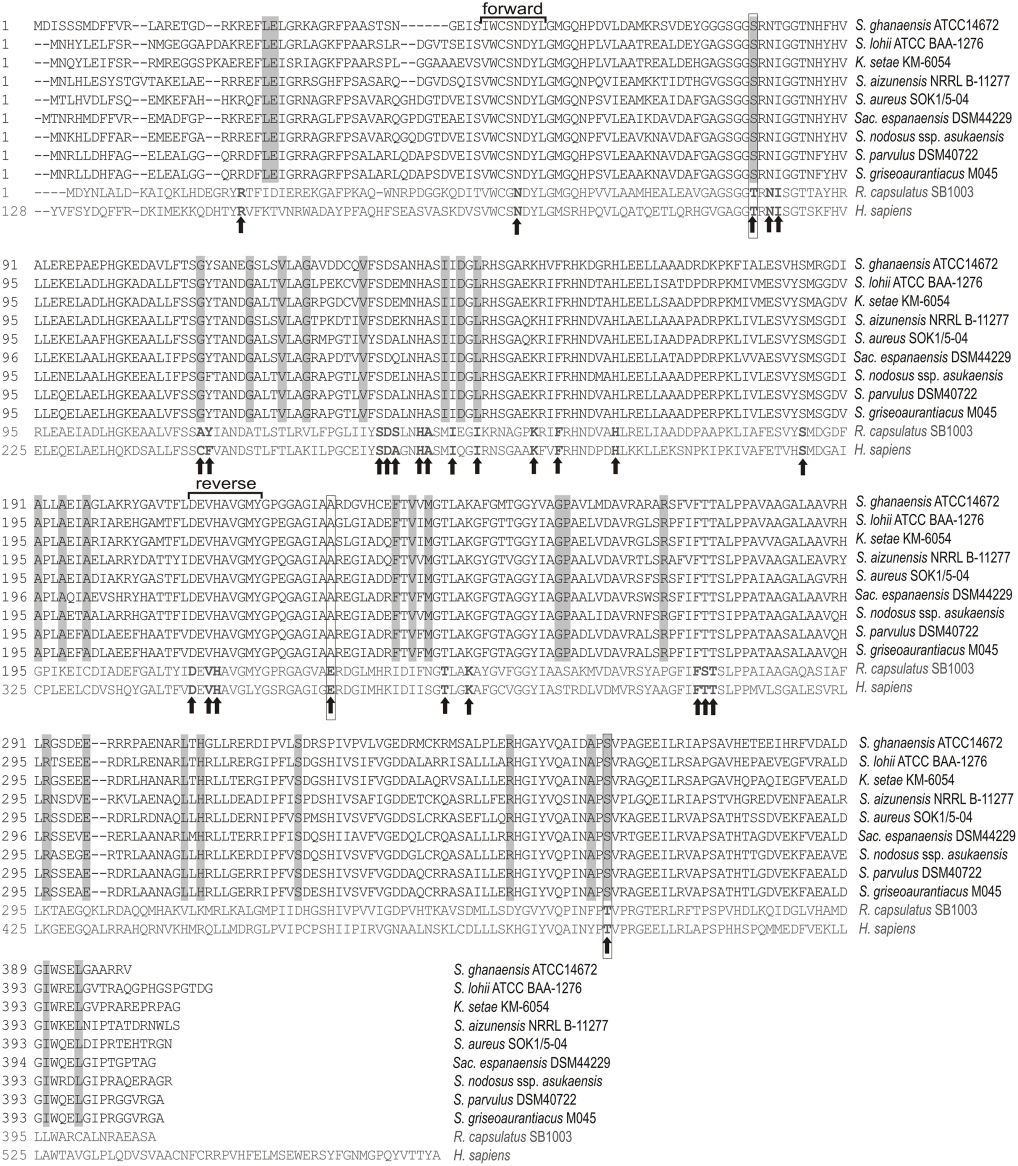
**Amino acid sequence alignment of antibiotic-related cyclizing 5-aminolevulinate synthases (ALASs; black) with representatives of standard proteobacterial and human ALAS (gray).** Original organisms abbreviated as follows *S. – Streptomyces, Sac. – Saccharothrix, K. – Kitasatospora, R. – Rhodobacter, H. – Homo*. Amino acid residues directly involved in binding of substrates and the PLP cofactor indicated in bold with arrows (Astner et al., 2005). Residues different from classical ALAS and strictly conserved in cALAS boxed gray and outlined in black when residues are directly connected to the enzyme activity. Conservative amino acid stretches used for the screening PCR method design are indicated.

Available actinomycete genomes were scanned for putative ALAS-coding regions using a standard BlastP algorithm with well characterized cALAS as queries (cut off set to 40% identity and 60% similarity). The protein sequences retrieved were inspected for conservation of ALAS-typical regions (**Figure 1**, Supplementary Table S3).

### Isolation of Actinomycete Strains and DNA Extraction

In total, 1,500 streptomycete-like bacterial strains were isolated from 53 environmental samples (Supplementary Table S1) from soils and sediments representing unique habitats around the world (Supplementary Table S2; includes strain isolation sources).

Samples were first homogenized by sieving through a 5 mm sieve then cultured according to Krištůfek et al. (2005). Where necessary, samples were stored for 1–2 days at 4°C. Serially diluted samples were plated onto R2A agar (BD Difco, Franklin Lakes, NJ, USA), McBeth–Scales starch-mineral agar or M2 agar (Shirling and Gottlieb, 1966) for isolation and culturing of streptomycete strains. The plates were then incubated in the dark at 28°C. Bacterial cultures were maintained long-term as spore suspensions in glycerol (15% v/v) at -80°C and/or freeze-dried. One group of strains had been isolated and characterized previously (Chroňáková et al., 2010), consequently we studied one of these in greater detail (*S. aureus* SOK 1/5-04, BCCO10_5, a new colabomycin E producer; Petříčková et al., 2014).

Several cooperating laboratories (see Acknowledgements, Supplementary Table S1) kindly provided sets of soil/sediment samples or streptomycete strains. Most of these are deposited in the Biology Centre Collection of Organisms, Culture Collection of Soil Actinomycetes in České Budějovice (BCCO^1^).

Genomic DNA was extracted using the Wizard^®^ Genomic DNA Purification kit (Promega, Madison, WI, USA) with slight modifications. We applied a combination of morphological comparison and genomic fingerprinting (BOX-PCR) to de-replicate strains isolated from the same environment.

### Screening for the cALAS Gene

ALAS-specific primers were designed based on the two most conservative amino acid stretches, i.e., VWCSNDYL and DEVHAVGMY (**Figure 1**), with respect to the strong GC preference on the third codon position typical for streptomycete coding regions. Two degenerative primers were designed: HemA1 (5′-GTS TGG TGY TCS/RGS AAC GAC TAC CT-3′) and HemA3 (5′-GTA CAT SCC SAC SGC GTG SAC CTC GCT-3′). Each sample (25 μL) contained 1x Fast Start PCR Master (Roche Applied Science, Penzberg, Germany), the primers (1.6 μM each) and 200 ng of template DNA. PCR reaction conditions were optimized using chromosomal DNA from previously characterized C_5_N-compound producers (*S. parvulus* Tü64, *S. bambergiensis* ATCC13879, *S. nodosus* sp. *asukaensis* ATCC29757) and with *S. coelicolor A3(2)* and *S. lividans* TK24 strains used as negative controls. Thermal cycling was performed as follows: Initial denaturation at 95°C for 2 min; followed by 30 cycles of denaturation (94°C/30 s), annealing (60–55°C/30 s; annealing temperature touchdown decreased by 0.5°C per cycle over the first 10 cycles, then kept at 55°C over the following 20 cycles), and extension (72°C/60 s; final extension 72°C for 7 min). The optimized PCR reaction yielded in a single band of 519 bp, with no variation in size. Amplified products were cleaned-up using the MinElute PCR Purification Kit (Qiagen, Hilden, Germany), eluted in 12 μL and sequenced using HemA1 primer. Relatedness of DNA sequences to ALAS genes was verified via a BlastX search of non-redundant protein sequences in GenBank.

### Phylogenetic Analysis

Nucleotide datasets were translated into amino acids and aligned in BioEdit 7.0.5.3 (Hall, 1999) using the ClustalW algorithm. All ambiguously aligned and unaligned sites were deleted. Edited datasets were then used in nucleotides to infer phylogeny. Bayesian inference was used to construct phylogenetic trees, computed using MrBayes 3.1.2 (Huelsenbeck and Ronquist, 2005) with five million generations (GTR + I + Γ model with gamma distribution in six categories). After deleting a burn-in, consensual topologies were computed from 45,000 sampled trees.

### Analysis of Secondary Metabolite Gene Clusters

Analysis of the putative biosynthetic genes clusters was performed using BlastX^2^. Prediction of PKS domains specificity was done with the help of AntiSMASH package (Medema et al., 2011; Blin et al., 2013).

### Extraction of Secondary Metabolites

Fermentation followed by extraction of secondary metabolites was performed as described by Petříček et al. (2006): After 3–4 days of cultivation (28°C, 200 rpm), mycelia were extracted with acetone and ethyl acetate. The post-cultivation media were extracted with ethyl acetate, too. Both final extracts were combined, dissolved in acetone, and analyzed. UHPLC-DAD-ToF-MS analyses were carried out with a Waters Acquity UPLC System (Waters) using the Acquity UPLC BEH C18 column under the conditions specified in Petříčková et al. (2014).

### Genetic Fingerprinting

DNA amplification and PCR fragment separation followed the procedure of Lanoot et al. (2004), using the BOXA1R primer 5′-CTACGGCAAGGCGACGCTGACG-3′ (Versalovic et al., 1994). Fingerprinting was performed as follows: PCR products (20 μL) were separated on 20 cm × 20 cm gels using 130 V, 400 mA for 240 min in 1 × TBE buffer. The gels were stained for 30 min in a 1 × TBE bath supplemented with ethidium bromide (1 mg L^-1^). A photograph of the gel was stored as TIFF file through a CCD coupled camera by using the Photo-Doc software (Vilber-Lourmat, France). Gels were imported into the GelCompar II software package (Applied Maths, Belgium), normalized and gel track densitometric curve similarity matrices calculated using the Pearson product-moment correlation coefficient. Dendrograms were then constructed using the complete linkage algorithm. Distinct BOX-PCR groups were defined using a 70% similarity limit.

### Isolate Identification

Representative ALAS gene-positive isolates were identified through 16S rRNA gene amplification using pA (5′-AGAGTTTGATCCTGGCTCAG-3′) and pH (5′-AAGGAGGTGATCCAGCCGCA-3′) universal bacterial primers and sequencing (pA, pH; Edwards et al., 1989). 16S rDNA amplification was performed using the protocol of Kyselková et al. (2012). Once amplified, the 16S rRNA genes were cleaned-up, sequenced then edited and assembled using Geneious 5.5.6 (Biomatters, Auckland, New Zealand). They were then compared against a type strain database (Ez-Taxon Database^3^; Chun et al., 2007) to retrieve the most relative species.

### Whole Genome Sequencing

Genomic sequence of BCCO10_981 strain was determined on the 454 GS Junior System (Roche). A total of 156, 457 reads comprising 70,475,443 bases were assembled using the Roche GS *de novo* assembler (version 2.7). The assembly yielded 1,532 contigs (unpublished).

### Accession Nos.

GenBank accession numbers for the resultant sequences are listed in Supplementary Tables S1 and S3.

## Results

### Amino Acid Sequence Identifiers of Cyclizing Form of ALAS (i.e., cALAS)

Using genetic data recently released about a number of evident C_5_N-compound producers (McAlpine et al., 2005; Ostash et al., 2007; Kalan et al., 2013; Zhang et al., 2013), we were able to align several typical cALASs with “classical” ALAS representatives. This helped us to recognize sequence specificities for “cyclizing” ALAS types (cALAS). The amino acid sequence displayed a number of residues specifically conserved in cALAS and these may be directly connected to the novel cyclization function of the enzymes (**Figure 1**). Some of the residues, conservatively substituted in cALAS, have previously been shown to be crucial for ALAS enzymatic activity, i.e., T^83^S, I^149^L/V, D^231^T, and T^365^S, numbered based on the accepted *Rhodococcus* structure model. Thr^83^ and Thr^365^ are localized directly in the catalysis site (Kaufholz et al., 2013). Thr^83^ is important for positioning of the succinyl-CoA terminal carboxyl, and also directs amino acid specificity of ALAS. However, in cALAS model enzyme from the asukamycin biosynthetic route, its substitution with Ser is crucial for the cyclization function (Petříček et al., 2006). The sequence markers identified can be used to distinguish between ALAS and cALAS.

### Identification of *hemA* Homologs in Genomic Data of Actinomycetes

**FIGURE 2:**
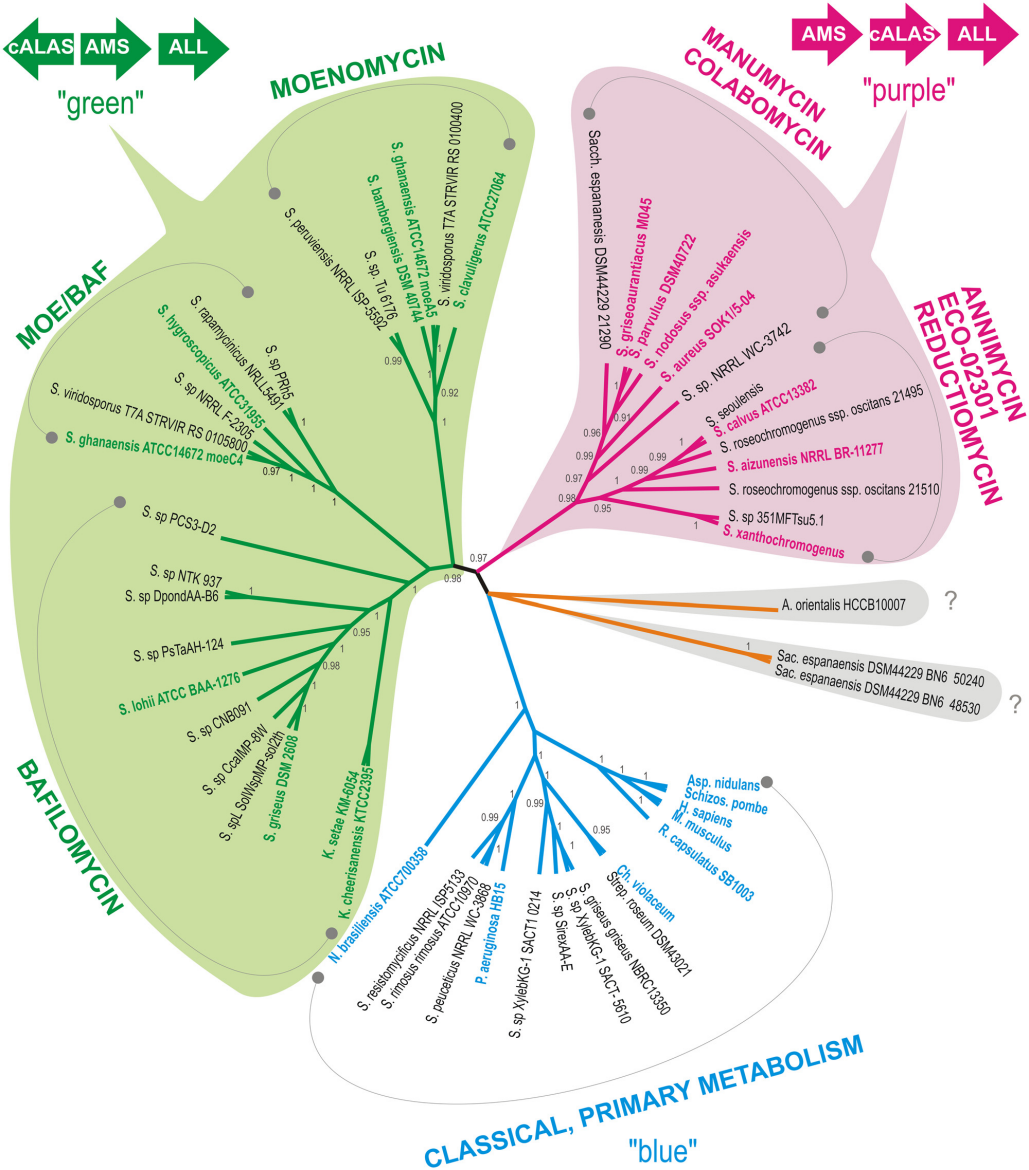
**Cladogram of *hemA* was constructed as specified in the Section “Materials and Methods,” posterior probability values are given when higher than 0.90.** All branches encompassing genes encoding putative cyclizing type of ALAS (cALAS) are color-shaded, types of secondary metabolites based on characterized producers are indicated in each branch. Reference sequences – genes of primary metabolism ALASs (“blue”) and of secondary metabolite-connected cALASs (“purple” and “green”), are shown in bold. Orange branch covers strains carrying cALAS-encoding *hemA* genes without a close phylogenetic relationship to any characterized producer strains. Original organisms are abbreviated as follows: S. – *Streptomyces*, K. – *Kitasatospora*, A. – *Amycolatopsis*, N. *– Nocardia*, Sac. – *Saccharothrix*, P. – *Pseudomonas*, Ch. – *Chromobacterium*, R. – *Rhodobacter*, Asp. – *Aspergillus*, Schiz. – *Schizosaccharomyces*, H. – *Homo*, M. – *Mus*. Organization of the C_5_N biosynthetic gene triad, encoding amide synthase (AMS), cALAS, and aminolevulinate ligase (ALL), conserved within the two major producer groups, designated as “green” and “purple,” is illustrated.

Sequences of all *hemA* gene homologs, identified by genome scanning of actinomycete genomic data using BLASTP search with AsuD2 cALAS as a query and listed in Supplementary Table S3, were added to the alignment and a cladogram constructed to illustrate phylogenetic relationships among the genes. Referential genes of the characterized producers (type producer strains) and a few representatives of primary metabolism *hemA* genes were also included. As expected, the cALAS-encoding representatives were clearly separated from “classical” ALAS. As C_5_N-carrying compounds display remarkable structural variability and diversity of relevant genetic information, we expected *hemA* genes evolution to follow that of entire biosynthetic gene clusters. Indeed, the genes show apparent clustering based on the type of C_5_N-containing compound produced (**Figure 2**). Two major producer groups were identified: The first, designated as “a purple group,” encompasses producers of simple linear polyketides and reductiomycin, which are characterized by head-to-tail organization of the C_5_N-encoding gene triplet *ams*-*hemA*-*all* and by direct attachment of the C_5_N unit to a polyketide chain. The second, “a green group,” represents producers of complex compounds such as sugar-containing polyketides or macrolides. In this case, the *hemA* gene is located upstream of the *ams-all* pair in the divergent orientation (**Figures 2** and **4**). Only two putative *hemA* loci of *Saccharothrix espanaensis* show different arrangement of the C_5_N-encoding genes (discussed later, **Figure 6B**). Contrary to original expectations, substantial fraction of identified genes (15%) clustered clearly with typical primary metabolism *hemA* representatives and their products did not show cALAS sequence characteristics.

In all identified genomic *hemA* homologs, the relevant genomic regions were analyzed for presence of putative secondary metabolites biosynthetic gene clusters using BLAST and AntiSMASH searches (see in Materials and Methods).

### PCR Screening of Natural Actinomycete Isolates for Presence of ALAS-Encoding Genes

**FIGURE 3:**
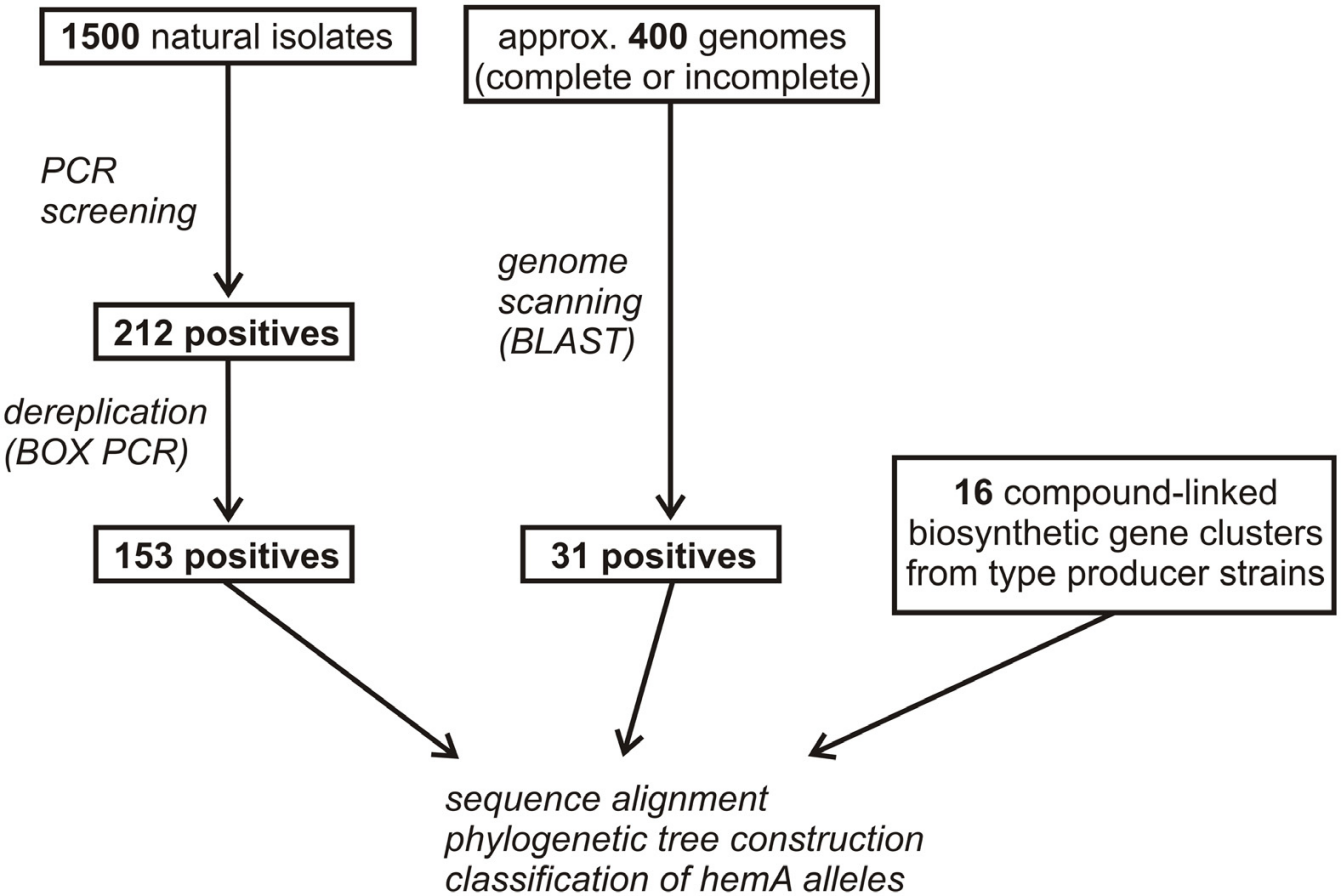
**Flowchart of *hemA* positive strains selection.** Numbers of screening-derived, genome scanning-derived, and referential producer strains are given.

**FIGURE 4:**
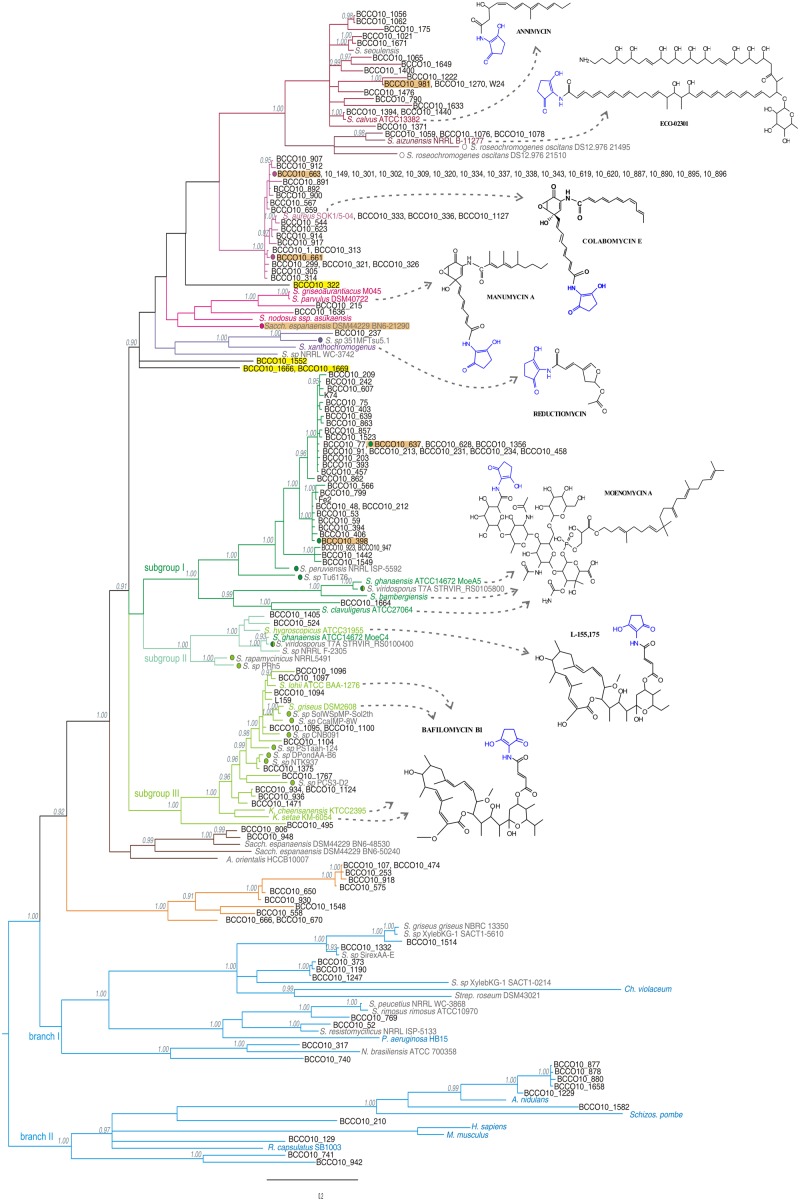
**Phylogenetic tree designed on the basis of the nucleotide sequence alignment of the *hemA* gene PCR fragments from the screening, calculated as specified in the Section “Materials and Methods.”** Posterior probability values are indicated in gray when higher than 0.90. The individual branches are differentiated by a color code corresponding to the type producers or reference sequences: producers of linear polyketides and reductiomycin in shades of purple, those of moenomycins or bafilomycins in greens, primary metabolism *hemA* genes in blue, and separate branches with no reference sequences in brown and orange, individually standing isolates are highlighted in yellow. Model structures of produced compounds are indicated for each branch. Sequences from characterized producers of C_5_N compounds and those of typical animal, fungal and bacterial ALAS genes are shown in colors (corresponds to **Figure 2**), genome-scanning matches are in gray, screening-based sequences in black letters. Color dots mark the strains with genetic information indicating production of similar compounds as a type strain of the branch. Isolates more closely characterized are highlighted in orange.

The PCR screening assay, described in the Section “Materials and Methods,” was designed to amplify both ALAS and cALAS-encoding *hemA* genes. It was applied to 1,500 new streptomycete-like isolates where 14% of strains were found positive, giving a single clear band of invariable size of 519 bp. In some other isolates, slightly smaller fragments were found. These were identified as fragments of putative *hemA*-related genes encoding 8-amino-7-oxononanoate synthases, which were not subjects of this work. All the *hemA*-positive isolates were dereplicated using BOX-PCR relatedness survey (see later) to eliminate possible multiple isolates. All 519-bp PCR fragments from 153 dereplicated positives were sequenced and shown to contain part of the *hemA*-homologous gene. These sequences, and the relevant fragments of the genome-scanning genes, were aligned both with the corresponding regions of cALAS-encoding *hemA* genes of type producer strains and with examples of classical *hemA* genes of various origins (Supplementary Table S3, **Figure 3**). A phylogenetic tree was constructed based on the alignment (**Figure 4**).

The majority of PCR sequences and genomic hits were tightly clustered within two major branches of known C_5_N compounds producers (green or purple, **Figures 2** and **4**). Few strains, which also carried genes with characteristics of cALAS-encoding *hemA*, formed two independent branches (orange and brown, **Figure 4**). Nineteen screening-based sequences, together with eight genomic sequences, were situated among typical, primary metabolism *hemA* genes of proteobacteria, bacilli, or fungi (blue groups, **Figure 4**). Functional and evolutionary aspects of these groups are discussed below.

### The “Purple Branch,” the “Head-to-Tail” Operon, the Producers of Linear Polyketides and Reductiomycin

Sixty-six natural isolates were clustered in the linear polyketides branch, together with six uncharacterized genome-based sequences. Thirty eight of the natural isolates formed a separate branch represented by a novel colabomycin E producer, *S. aureus* SOK1/5-04 (BCCO10_5), which was identified by the genetic screening method described in this work and recently studied in detail by our group (Petříčková et al., 2014). The next two isolates occurred in the manumycin-producer branch (BCCO10_215 and BCCO10_1636; isolated from Sokolov colliery spoil heaps and Mt. Cameroon foggy forest soil, respectively). Twenty-one isolates and three genomic sequences were localized among the PKSI-synthesized polyketide producers, ECO-02301 and annimycin. The reductiomycin branch encompassed one isolate (*Amycolatopsis thailandensis* BCCO10_237) and two genomic sequences. Four remaining sequences formed individual branches not tightly associated with known producers (**Figure 4**, in yellow). BCCO10_0322, BCCO10_1552, BCCO10_1666 and BCCO10_1669 may represent producers of either novel compounds or producers of previously characterized C_5_N-polyketides with genetic data lacking.

To verify the metabolite-based clustering, two strains, BCCO10_661 and BCCO10_663, of the colabomycin group were subjected to UPLC-MS (ultra-performance liquid chromatography tandem mass spectrometry) analysis of secondary metabolites and their putative biosynthetic gene clusters were partially sequenced and compared to that of colabomycin E producer. Both isolates shared the colabomycins production profile of *S. aureus* SOK1/5-04 and also their gene clusters organization was identical (not shown). Most of the strains of the colabomycin group originated from the same habitat, the Velká Podkrušnohorská spoil heap in the Sokolov brown coal mining area, Czech Republic, though from several distant localities differing mainly in succession age and streptomycete community composition (Chroňáková et al., 2010). Only two were isolated from different habitats: BCCO10_1127 from an agricultural field in Bad Lauchstädt, Germany, and BCCO10_544 from the Domica Cave river sediment, Slovakia (Supplementary Tables S1 and S2). Strains carrying *hemA* sequences related to colabomycin E branch isolated from similar habitats (H33-35, H37, H39-40, Supplementary Table S2) belonged to five phylogenetic clades according to Labeda et al. (2012) and they differed also by BOX-PCR groups (Supplementary Table S1, Supplementary Figure S3). These findings may support the evidence for lateral transfer of partial or entire biosynthetic gene clusters in streptomycetes in soil recorded previously (Egan et al., 1998; Deng et al., 2011).

Next, *S. graminilatus* BCCO10_215, clustering tightly with manumycin and asukamycin producers was proved to produce manumycin D and manumycin A in the ratio of 2,5:1, both compounds found also in the characterized producer *S. parvulus* Tü64 where manumycin A is the most prevalent (data not shown). We have also attempted to isolate annimycin-type compounds from the *S. sioyaensis* BCCO10_981 originated from the coal-mining recultivated soil in Wyoming, USA, as suggested by the phylogenetic data. No annimycin-type compounds were identified in the metabolic extracts of the strain under various cultivation conditions. Therefore, the genome of the strain was sequenced using 454 pyrosequencing (Roche) and the *hemA* locus identified (GenBank Accession No. KR364704). The *in silico* analysis revealed a biosynthetic gene cluster highly similar (more than 90% identity) to that of the annimycin producer, *S. calvus* ATCC13382. However, one of the *ann* genes, encoding the antibiotic transporter/resistance protein, is damaged by several deletions and insertions. We believe that this abolishes production of annimycin-type compounds in the *S. sioyaensis* BCCO10_981.

### The “Green Branch,” with “Head-to-Head” Organization of C_5_N Biosynthetic Genes, the Producers of Isoprenoids and Glycosylated Macrolides

The green branch (**Figures 2** and **4**) can be divided into three subgroups: the subgroup I comprising 38 isolates and three genomic sequences clustered with three moenomycin producers (Ostash and Walker, 2010); the subgroup II including two isolates, four genomic sequences, and *S. hygroscopicus* ATCC31955 (Kim et al., 2012) with *S. ghanaensis* ATCC 14672 (the *moeC4* gene), the type producer strains; and the subgroup III of 13 isolates and eight genomic sequences localized around four bafilomycin producers (Ichikawa et al., 2010; Hwang et al., 2013, 2015; Zhang et al., 2013).

**FIGURE 5:**
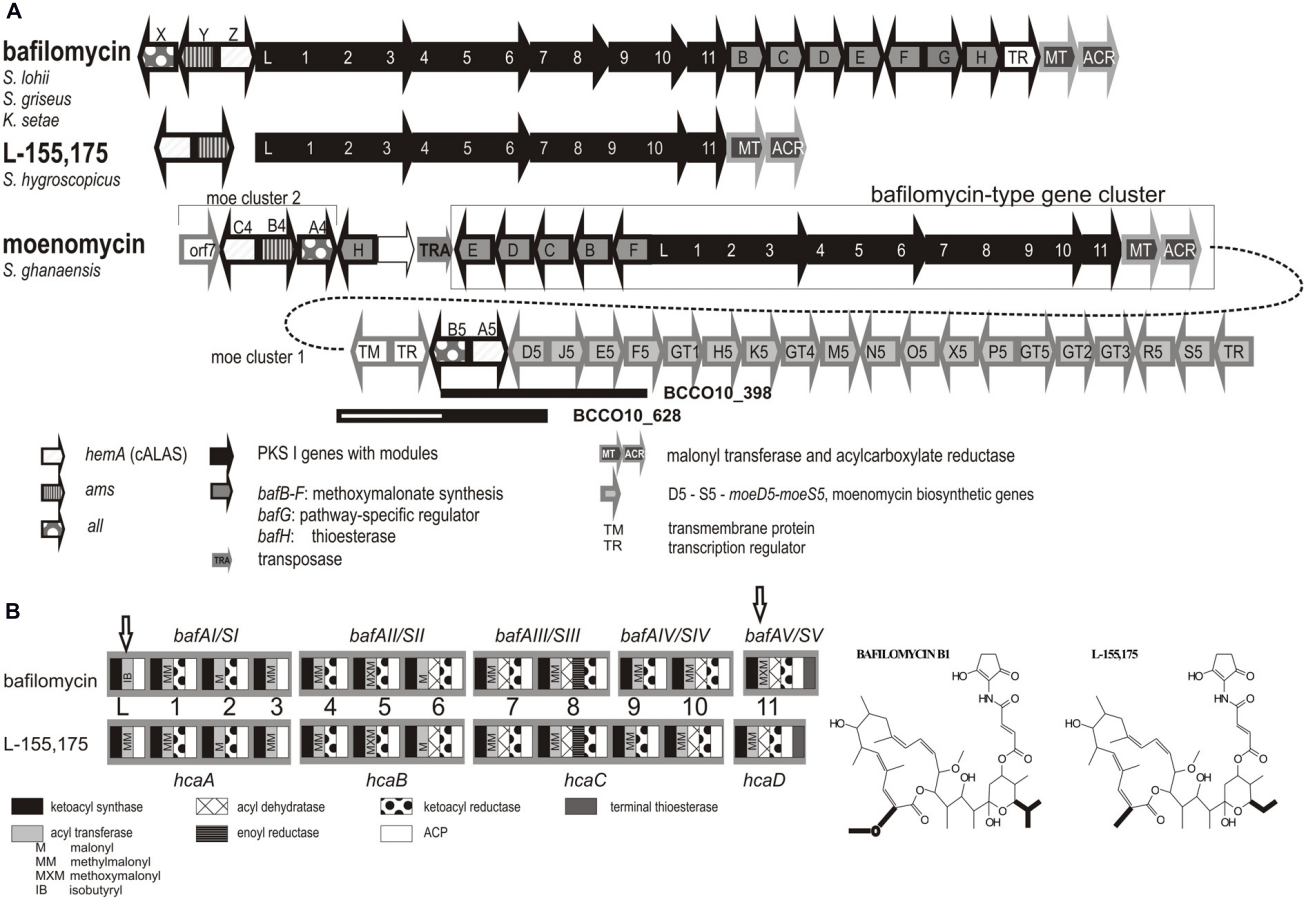
**Moenomycin and bafilomycin biosynthetic genes clusters. (A)** Rearrangement of the moenomycin- and bafilomycin-type clusters in some producers of moenomycin. PKS genes are shown in black, modules indicated by numbers. Bafilomycin biosynthetic genes outlined in black (PKS genes in filled black, tailoring genes filled gray), moenomycin in gray. Homologous regions identified in isolates Nos. BCCO10_398 and BCCO_628 are shown as black rectangles. **(B)** Domain organization within PKS genes – comparison of closely related clusters encoding biosynthesis of bafilomycin B1 and L-155,175. Specificity of AT domains, corresponding to the differences in the metabolite structures, highlighted in bold, are included (different domains indicated with arrows).

Most of the putative moenomycin-type isolates formed a separate branch (subgroup I), with only one strain clustered tightly with the type producer strains. Therefore, two strains *S*. *aurantiacus* BCCO10_398 and *S*. *ederensis* BCCO10_628 were more closely characterized genetically. Short genomic fragments of their *hemA* loci were cloned and sequenced. Both showed similar gene organization as *moe* cluster 1 of the moenomycin producer *S. ghanaensis* (Ostash et al., 2007), with a short non-homologous region in BCCO10_628 (homologous parts indicated as black rectangles in **Figure 5A**). We suppose that at least majority of the subgroup I strains produces moenomycin-type compounds.

Similarly, the subgroup III seems to include only putative bafilomycin compounds producers clustering tightly with 4 type producers of bafilomycin-type compounds. The *hemA* sequences in this subgroup are highly similar, with only BCCO10_495 staying a bit separately. Some of the novel isolates (all six ranging between Nos. BCCO10_1094-1104) share a geographical origin with one of the bafilomycin producer *S. lohii* ATCC BAA-1276, isolated from beach sands in Papua New Guinea (Zhang et al., 2013). In contrast, our strains were isolated from ambrosia beetle galleries and were classified as three different species (Supplementary Tables S1 and S2). The origins of remaining eight positives are diverse. Again, these strains are phylogenetically distant and do not display BOX-PCR genomic similarity (Supplementary Figure S3; Supplementary Table S1). Eight genome-project sequences mostly from insect-associated or marine streptomycetes also occurred in the branch.

Interpretation of the subgroup II was not clear at the first sight: It contained the *hca1* (*hemA*) gene from a producer of a bafilomycin derivative L-155,175 (Kim et al., 2012), but also the *moeC4* of moenomycin gene cluster 2 of *S. ghanaensis* ATCC 14672 (Ostash et al., 2007). This extensively studied strain was reported to contain two physically distant C_5_N-encoding loci connected with moenomycin production (**Figure 5A**): The major *moe* biosynthetic cluster 1 carries impaired genetic information for C_5_N biosynthesis, with a truncated *all*, missing *ams* and non-functional *moeA5* (*hemA)* gene. This is suppressed by *moe* cluster 2 elsewhere in the genome - *moeA4, B4, C4* (Ostash et al., 2007). We found identical situation also in the sequenced genome of *S. viridosporus* T7A and in the genomic DNA of the moenomycin producer *S. bambergiensis*, previously analyzed in our laboratory (data not shown). Whereas the *hemA* genes of *moe* cluster 1 (i.e., *moeA4*) fit into the subgroup I, the genes from the cluster 2 (i.e., *moeC4)* are phylogenetically more related to *hca1* of *S. hygroscopicus* ATCC31955, the L-155,175 producer (Kim et al., 2012). To clarify these findings we inspected the neighborhoods of *S. ghanaensis moe* cluster 2. Indeed, in the available gapped genomic sequence of *S. ghanaensis* we identified a putative L-155,175-type biosynthetic gene cluster just next to the *moe* cluster 2. The type I polyketide synthases encoded there showed approx. 90% amino acid sequence identity with *hcaA-D* genes products. Also, the PKS module organization and predicted specificities of their acyl transferase domains were the same (**Figure 5**). Unlike the L-155,175 *hca* cluster, however, *S. ghanaensis moe* cluster 2 locus carries all putative methoxymalonyl-CoA biosynthetic genes (*bafB-F*) as in subgroup III bafilomycin producers, and intact C_5_N-encoding genes. A putative transposase gene is inserted between the annotated *moe* cluster 2 and the L-155,175 cluster-like region (**Figure 5**).

### The Orange and Brown Branches – Still Secondary Metabolite Related?

Both the orange and brown branches were clearly separated from any other (**Figure 4**). The brown branch contained two *hemA* sequences (BCCO10_806 and BCCO10_948) from natural strain isolates and three from genomic data, while the orange branch included 11 sequences from various biotopes and were assigned to six different streptomycete species (Supplementary Tables S1 and S2). The amino acid sequences encoded by the *hemA* PCR fragments indicate that the majority of residua typical for cALAS, including the key T83S mutation, are conserved in the brown branch representatives, though some were missing in members of the orange branch (**Figure 1**, Supplementary Figure S4).

**FIGURE 6:**
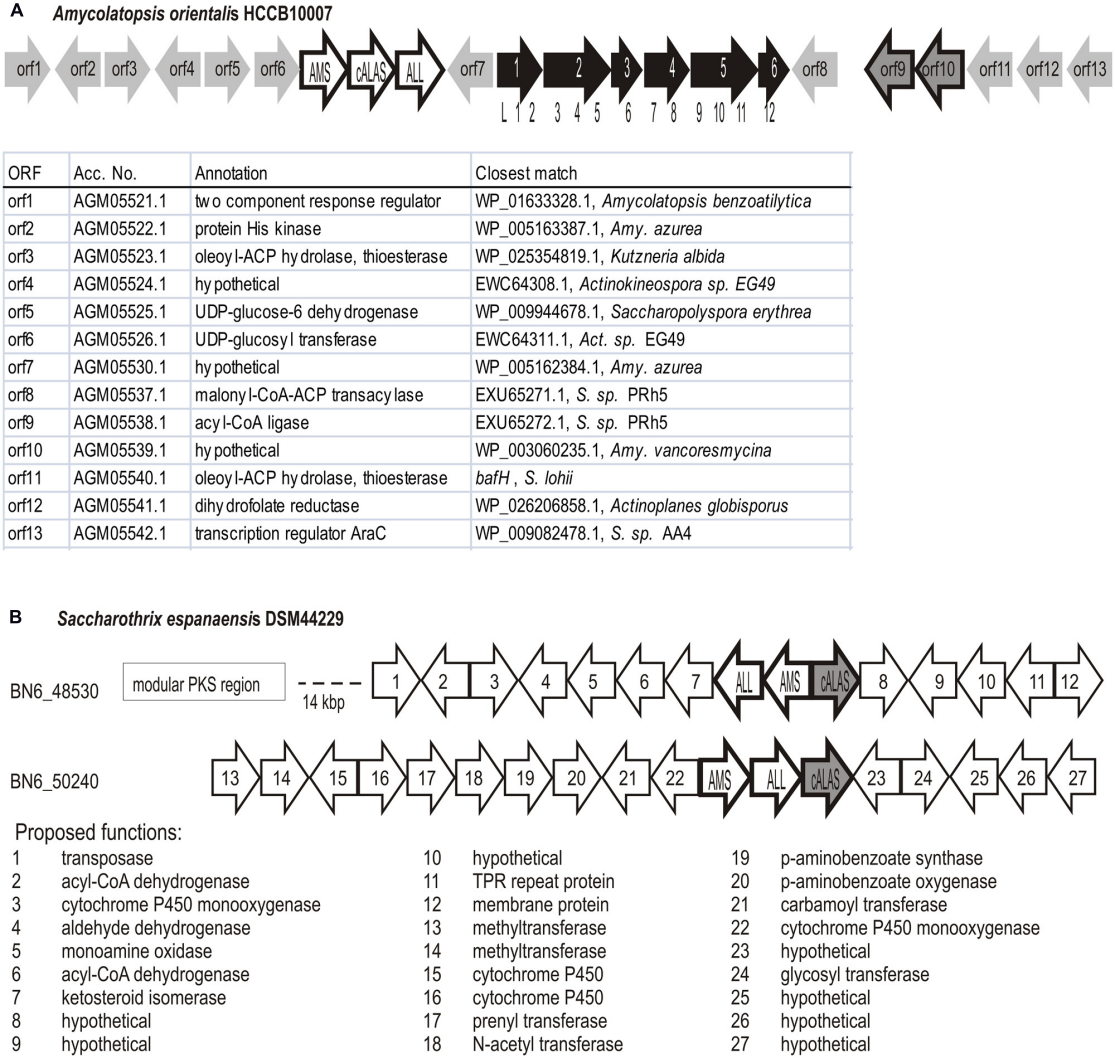
**Genetic loci containing C_5_N-like operons in *Amycolatopsis* and *Saccharothrix* of the orange branch.** The C_5_N-like operons genes marked AMS, cALAS, ALL. **(A)** Potential polyketide encoding genetic cluster in *Amycolatopsis orientali*s HCCB10007. Modular PKS genes shown filled with black, module distribution among the genes indicated by numbers below. The remaining ORF list is shown below the scheme. **(B)** *Saccharothrix espanaensis* DSM44229, two non-manumycin-type C_5_N operons (BN6_48530 and BN6_50240 indicate Gene Bank Accession Nos. of relevant *hemA* genes) and their genetic context.

The brown branch contained *Amycolatopsis orientalis* sequences and two *hemA* genes from *Saccharothrix espanaensis* DSM 44229 (ORFs BN6_48530 and BN6_50240*).* Interestingly, both “brown” natural isolates (BCCO10_948 and BCCO10_806) were also identified as *S. espanaensis*, though they originate from geographically distant localities (Tennessee re-cultivation area and Sokolov colliery spoil, Czech Republic, respectively). Organization of the C_5_N-encoding genes in this group was not uniform (**Figure 6**), but they were always surrounded by putative secondary metabolism genes. In *Amycolatopsis* and *Saccharothrix* BN6_48530 loci, PKS I genes were found in the *hemA* neighborhoods (**Figure 6B**).

### General Observations in *hemA* Genes Putatively Encoding Cyclizing ALAS

All genomic data-derived *hemA* genes, showing characteristics of the cyclizing type, were routinely found within a triplet of C_5_N-biosynthetic genes (*ams, hemA, all*) that was located in a putative secondary metabolite biosynthetic gene cluster. Within the major groups of strains, defined by the *hemA* phylogeny, these gene clusters typically showed high similarity to the gene clusters of type producer strains (conformity with the type producers indicated in **Figure 4** as colored dots in front of the strain names). This applies also to the few natural isolates with more detailed genetic analysis of the *hemA* loci. In majority of these metabolite-related groups, the homologous biosynthetic gene clusters share the organization of the genes and most of the differences are due to more or less numerous point mutations in individual biosynthetic, regulatory and transport genes. The only exception is the group of manumycin-type metabolites. Here, in most of the known cases, the ordering of individual biosynthetic genes or operons within the clusters differs substantially, though the metabolites share identical core structures. Also, phylogenetic relationships of the corresponding *hemA* genes are less tight than in other groups.

### The Blue Branches – The “Black Box”

Sequences in the blue group were not clustered in any secondary metabolite-related group and were closely related to non-cyclizing primary-metabolism *hemA* genes. It encompassed eight genome project-derived sequences and 19 screening fits (seven *Streptomyces* and one *Nocardia* species; **Figure 4**; Supplementary Table S1) that formed the two least homogenous branches of all. They do not show typical sequence characteristics of cALAS-encoding genes and have slightly lower GC% contents than usual in streptomycetes. The blue branch I, with the closest match to *Chromobacterium violaceum*, included the GC-rich sequences, > 62%; whereas the blue branch II more closely matched fungi and included the sequences with GC% < 62. The GC% trend matched those of the closest matching *hemA* sequences, used as references in each branch (Supplementary Table S3).

**FIGURE 7:**
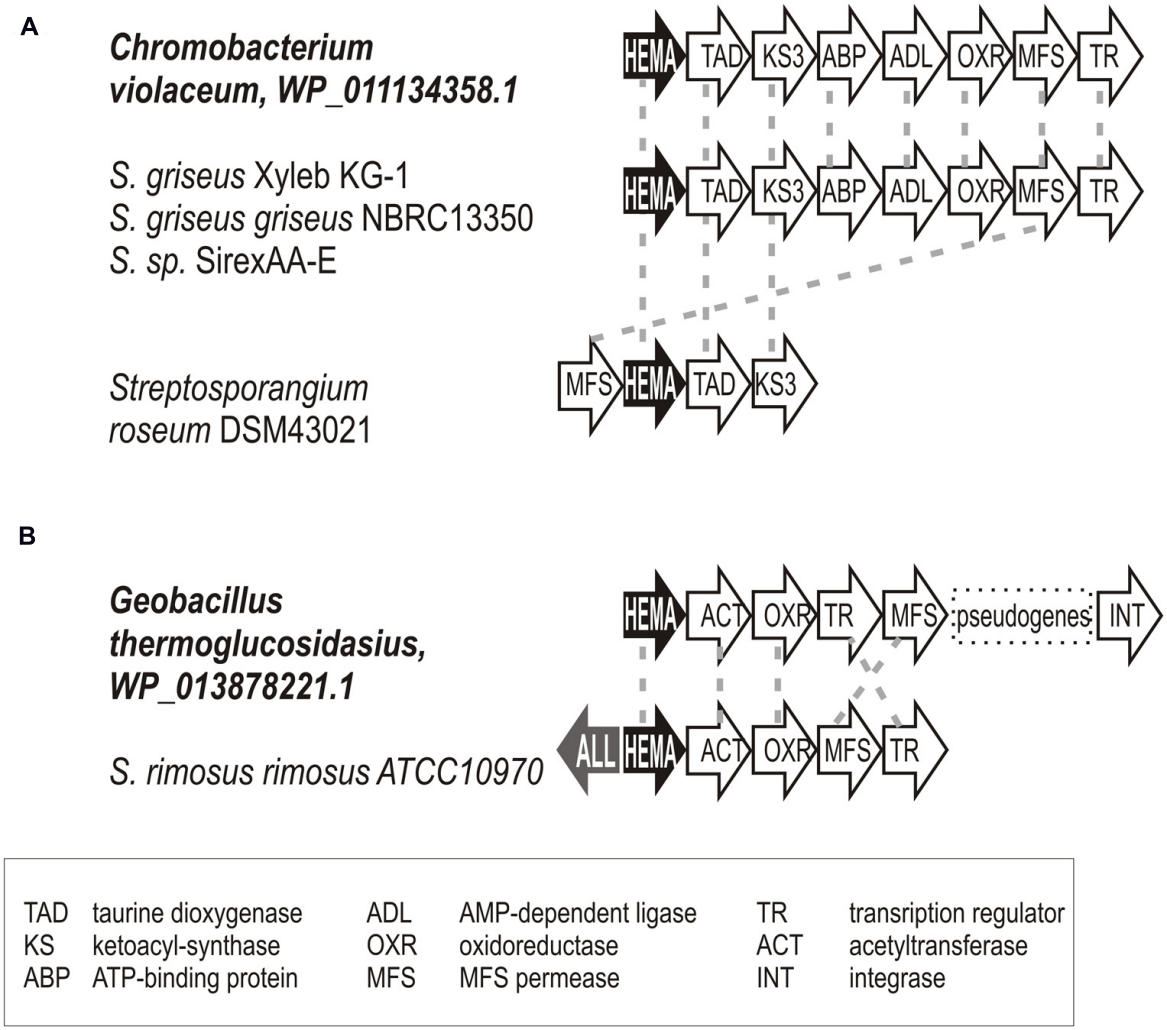
**Examples documenting the horizontal gene transfer of *hemA* gene loci between proteobacteria or bacilli and actinomycetes.** Relevant genetic loci of putative donors and acceptors are shown; gene homology indicated by dashed gray lines. Gene annotations are given in the figure bottom. **(A)** *Chromobacterium violaceum* **(B)** *Geobacillus thermoglucosidasius*.

ALAS-encoding genetic loci in representatives derived from genome projects indicated no physical association with any C_5_N-type operon. Their genetic context was not conserved and their secondary metabolism function is speculative. Attempts to identify the closest homologs of the blue group sequences indicate that the blue branch II sequences show stronger match to proteobacterial *hemA* genes or to those of *Bacilli*. The branch II sequences are more similar to *hemA* of *Aspergillus.* In some cases, we documented several kbp regions surrounding the *hemA* gene that were identical to their non-actinomycete homologs (see **Figure 7** for examples).

### Isolate Identification and Genome Relatedness

To underpin genetic diversity of environmental strains coming into screening procedure, 234 were subjected to a relatedness survey. Genomic fingerprinting indicated high genetic diversity. The BOX-PCR similarity profile divided the environmental isolates into 184 distinct groups, using the Pearson product moment correlation coefficient threshold of 70% (Supplementary Figure S3), of which 147 were unique. The most abundant BOX-PCR groups were clusters B10 and B34, both comprising five strains from two closely related habitats. With the exception of groups B11, B12, B28, and B36, isolates from one BOX-PCR group were usually assigned to the same species or clade (Supplementary Table S1).

The 16S rRNA genes of 131 isolates were sequenced with the aim to characterize most of BOX-PCR groups (at least those comprising of more than one strain). Most environmental isolates had identical or almost identical sequences (98-100% pairwise sequence similarity) to species of genus *Streptomyces* – 124 strains, *A. thailandensis* – 1, *Kitasatospora atroaurantiaca* – 1, *K. gansuensis* – 1, *Lentzea violacea* – 1, *Nocardia nova* – 1 and *Saccharothrix espanaensis* – 2 (Supplementary Table S1). Forty-nine species have been recognized within the *Streptomyces*, of which *S. aurantiacus, S. aureus*, and *S. ederensis*, along with *S*. *griseus*, a group of taxonomically related species, are the most prevalent (comprising 9, 12, 11, and 16 strains, respectively).

Apparently, in four BOX-PCR groups (B11, B12, B28, and B36) a discrepancy between 16S rDNA phylogenetic clade assignment and BOX-PCR grouping was observed. The same BOX-PCR groups contained more or less phylogenetically related species (e.g., *S. aureus* and *S*. *olivochromogenes* in group B11; *S*. *phaeochromogenes* and *S*. *olivochromogenes* in group B12; *S*. *scabiei* and *S*. *lavendulae* in group B28; *S*. *prunicolor* and *S*. *griseus* in group B36). Additionally, species were often represented by several ecotypes (e.g., more than one BOX-PCR group has been assigned to the same species). This was more the rule than the exception, even when strains originated from the same habitat (e.g., *S. aurantiacus* and *S. ederensis* from habitat H32). This finding supports the idea that genomic rearrangements and other kinds of genetic flow are occurring in natural strains frequently and should be taken into account.

## Discussion

In this work we showed the independent evolution of aminolevulinate synthase genes in actinomycetes, especially of their newly described, cyclizing, secondary metabolism-connected variant encoding cALAS. These represent a key enzyme in the C_5_N unit formation. Together with accompanying AMS and acyl-CoA ligase they direct biosynthesis and attachment of the functional moiety to different classes of bioactive secondary metabolites of actinomycetes. Thus, the *hemA* gene may be used as a genetic tag for putative producers of C_5_N carrying-compounds. This variable group contains secondary metabolites with pharmaceutically attractive potential, such as alternatives for β-lactams, anti-cancer compounds, and immunomodulatory molecules. Definition of metabolite-specific signatures for *hemA* gene sequences may serve as a useful tool in identifying novel producers of known compounds or their new derivatives, or perhaps in identifying putative producers of novel C_5_N compound classes. Moreover, our data illustrate the distribution and relative frequency of relevant biosynthetic genes in genomes of actinomycetes. We provide several examples suggesting horizontal gene transfer within actinobacterial communities in particular biotopes, and genetic evidence of gene transfer between bacterial and fungal genera.

Relatively short fragment-sequences of aminolevulinate synthase genes were shown to provide sufficient genetic information to differentiate between putative producers of the individual C_5_N compounds. The phylogenetic tree of short *hemA* fragments showed clear clustering of sequences corresponding to compounds. Of the 153 positives analyzed (10% of all screened strains) 88% appear to follow cALAS-specific sequence conservation. Most of these fit into the branches of known type producers (78% of all positives). The remaining 12% of cALAS-like *hemA* homologs from the screening, together with 9% from genomic matches, were independent of known producer branches (brown and orange branches in **Figure 4**). These displayed most of cALAS-specific sequence features; hence we can speculate that they form the C_5_N or similar cyclic units. From the genome data available in the brown group we may also observe, that *hemA* genes are again accompanied with both *all* and *ams* genes here and lay within putative secondary metabolites biosynthetic genes. These represent the hottest candidates for novel types of C_5_N-containing bioactive compounds producers.

Genomic data suggest that, rarely, streptomycete genomes may carry two putative biosynthetic gene clusters for C_5_N metabolites of two different classes, as it is in *S. ghanaensis* ATCC14672 and *S. viridosporus* T7A. The dysfunctional C_5_N-encoding locus of one is suppressed by the products of the other, located in a different gene cluster.

Unexpectedly, as high as 12% of screening sequences (and 24% of those from genomic projects) appear to encode non-cyclizing, “classical” ALAS, though this enzyme is not needed in the primary metabolism of actinomycetes as they form 5-aminolevulinate by the different, C5 pathway from glutamate (Petříček et al., 2006). Neither we have observed any obvious connection to secondary metabolism; no conservative, typical secondary metabolism genes were found in the *hemA* loci. On the other hand, the genetic data provide evidence of intergeneric horizontal gene transfer in soil microorganism communities, even between phylogenetically distant microorganisms, Proteobacteria, Firmicutes, and Actinobacteria, inhabiting the same biotope. This supports recent theories of a horizontal gene transfer between quite distant bacterial phyla (Shintani et al., 2014) or even crossing borders between kingdoms of Bacteria and Fungi, which is much less well understood (Schmitt and Lumbsch, 2009). Whether these “stolen” *hemA* genes are functional in actinomycetes remains unclear. We can hypothesize, however, that they were used as a starting material for cALAS evolution after their HGT into *Streptomyces*.

Horizontal gene transfer is also observable in cALAS-encoding *hemA* genes. For example, groups of *hemA*-positive isolates fitting to the same metabolite production group (colabomycin, moenomycin, e.g.), which originate from the same biotope, are often classified into different *Streptomyces* species. This is an important point to take into account when developing screening protocols in the search for novel producers of specific compounds as increased geographic and biotope diversity increases the chances of identifying new producers. Taxonomy data appear to be much less important. Huge parts of chromosomes and giant plasmids are traded within the actinomycete population in individual biotopes (Doroghazi and Buckley, 2010), and this was many times suggested also by our data, e.g., in the set of putative bafilomycin producers. The type strain, *S. lohii* ATCC BAA-1276, was isolated by a Chinese group from a sandy beach in Papua New Guinea (Zhang et al., 2013). Our own six isolates, isolated from ambrosia beetle galleries in Papua New Guinea, carry highly homologous *hemA* genes, though taxonomically they are classified as at least three different, non-related species. Of the habitats studied, a number display high genetic variability resulting in diverse secondary-metabolite production, e.g., Sokolov colliery spoil provided strains possessing *hemA* genes related to manumycin and colabomycin linear polyketides, moenomycin, as well as unknown orange and brown clusters.

## Conclusion

Our work proved the hypothesis of correlation between the secondary metabolism-connected *hemA* gene sequences and a type of the C_5_N-containing metabolite synthesized. This specifically concerns *hemA* alleles encoding cyclizing type of aminolevulinate synthase, as a part of the C_5_N-encoding gene triad. Therefore, phylogenetic analysis of *hemA* genes may assist in the search for novel producers of bioactive C_5_N compounds, as in the case of previously characterized producer of novel colabomycin E, *S. aureus* SOK1/5-04 (Petříčková et al., 2014). The developed PCR screening technique displays high sensitivity and specificity, and is simple, fast, and cheap. Moreover, the presented data imply frequent horizontal transfer of genes and entire clusters within actinomycete populations: Phylogenetically distant streptomycetes of similar geographical origin were shown to carry identical or highly similar *hemA* genes (bafilomycin producers from Papua New Guinea) and to carry the same biosynthetic gene clusters (colabomycin or moenomycin producers from Sokolov). On the other hand, less frequent *hemA* genes of non-cyclizing type were not associated with any secondary metabolism genes, showed lower GC contents, and sometimes, obvious homologies with putative donors in *hemA* chromosomal loci were found. This may suggest more recent horizontal gene transfer event from lower GC donors, which use *hemA*-encoded enzymes in their primary metabolism. As the gene is not essential for primary metabolism of actinomycetes, we may imagine that a similar event could earlier serve as a starting material for evolution of the novel biosynthetic capability of the acceptor – novel subunit was formed and subsequently attached to different metabolic cores.

## Author Contributions

KP planned the experiments, conducted the screening, sequencing and genome scanning, analyzed and interpreted the data, and prepared the manuscript; AC planned and performed phylogenetic analyses, made the whole-genome sequencing, and prepared the phylogenetic parts of the manuscript. TZ isolated genomic DNAs, performed the genetic screening and analyzed the secondary metabolite extracts. TC designed, performed, and interpreted phylogenetic analyses. SP made fermentations and prepared secondary metabolite extracts. MP and VK supervised the experiments, MP also contributed to sequencing and data analysis, and VK collected and isolated all the novel strains.

## Conflict of Interest Statement

The authors declare that the research was conducted in the absence of any commercial or financial relationships that could be construed as a potential conflict of interest.

## Footnotes

1 www.actinomycetes.cz

2 http://blast.ncbi.nlm.nih.gov

3 http://www.eztaxon.org
