# Supplementary material for: Evolution of cyclizing 5-aminolevulinate synthases in the biosynthesis of actinomycete secondary metabolites: outcomes for genetic screening techniques
Source: Front Microbiol. 2015 Aug 5;6:814. doi: 10.3389/fmicb.2015.00814 (PMC4525017; doi:10.3389/fmicb.2015.00814)
Supplement: Supplementary file 1 [file Presentation_1.PDF]

SUPPLEMENTARY MATERIALS

Supplementary Table 1. List of environmental strains positive in PCR screening for *hemA* gene presence, and their characteristics. Few negative strains (13) are also included. Taxonomic assignment, sequence similarity, and Accession Nos. of 16S rRNA and *hemA* genes are given. Strains are grouped by BOX-PCR similarity groups (Supplementary Fig. 2) and phylogenetic relatedness of *hemA* gene sequence to sequences of reference producer strains (Fig. 4 and Supplementary Table 3).

Strain No.	Taxonomic assignment (respective 16S rRNA group) according to EzTaxon	Sequence similarity (%)	Phylogenetic clades**	Accession No. (16S rRNA gene)	Box-PCR group	PCR detection of *hemA* gene	Accession No. (*hemA* gene)	Phylogenetic relatedness of *hemA* gene	Habitat code
**BCCO 10_375**	*S. sp.*	-	nd	nd	BOX1	+	nd		H35
**L159***	*S. bacillaris*	100.00	nd	KP718510	BOX1	+	KP404554	bafilomycin	H51
**BCCO 10_85**	*S. sp.*	-	nd	nd	BOX10	+	nd		H32
**BCCO 10_93**	*S. sp.*	-	nd	nd	BOX10	+	nd		H33
**BCCO 10_209**	*S. phaeochromogenes*	99.86	18	KP718586	BOX10	+	KP404457	moenomycin	H32
**BCCO 10_213**	*S. sp.*	-	nd	nd	BOX10	+	KP404557	moenomycin	H32
**BCCO 10_231**	*S. ederensis (S. umbrinus)*	100.00	18	KP718530	BOX10	+	KP404558	moenomycin	H32
**BCCO 10_619**	*S. olivochromogenes*	98.72	20	KP718582	BOX11	+	KP404576	colabomycin	H39
**BCCO 10_620**	*S. olivochromogenes*	98.72	20	KP718583	BOX11	+	KP404577	colabomycin	H39
**BCCO 10_3**	*S. sp.*	-	nd	nd	BOX11	+	nd		H39
**BCCO 10_663**	*S. aureus*	99.51	26	KP718503	BOX11	+	KP404578	colabomycin	H34
**BCCO 10_76**	*S. phaeochromogenes*	100.00	18	KP718585	BOX12	+	nd		H32
**BCCO 10_130**	*S. sp.*	-	nd	nd	BOX12	+	nd		H34
**BCCO 10_132**	*S. olivochromogenes*	98.72	20	KP718581	BOX12	+	nd		H34
**BCCO 10_301**	*S. sp.*	-	nd	nd	BOX13	+	KP404568	colabomycin	H34
**BCCO 10_337**	*S. aureus* SOK8/14-05	98.96	26	EU098044	BOX13	+	KP404573	colabomycin	H34
**BCCO 10_309**	*S. aureus*	98.94	26	KP718499	BOX14	+	KP404570	colabomycin	H34
**BCCO 10_320**	*S. sp.*	-	nd	nd	BOX14	+	KP404571	colabomycin	H34
**BCCO 10_917**	*S. sp.*	-	nd	nd	BOX15	+	KP404503	colabomycin	H34
**BCCO 10_1525**	*S. griseus subsp. griseus (S. griseus)*	99.57	112	KP718559	BOX15	+	nd		H28
**BCCO 10_253**	*S. griseus subsp. griseus (S. griseus)*	99.58	112	KP718557	BOX16	+	KP404462	orange cluster	H33

**BCCO 10_304**	*S. sp.*	-	nd	nd	BOX16	+	nd		H34
**BCCO 10_511**	*S. griseorubiginosus (S. griseus)*	99.58	7	KP718554	BOX16	+	nd		H12
**BCCO 10_575**	*S. griseorubiginosus (S. griseus)*	99.51	7	KP718555	BOX16	+	KP404479	orange cluster	H43
**BCCO 10_891**	*S. kanamyceticus*	98.92	nd	KP718565	BOX18	+	KP404497	colabomycin	H33
**BCCO 10_914**	*S. griseorubiginosus (S. griseus)*	99.58	7	KP718556	BOX18	+	KP404502	colabomycin	H33
**BCCO 10_131**	*S. sp.*	-	nd	nd	BOX19	+	nd		H34
**BCCO 10_298**	*S. aureus*	98.86	26	KP718498	BOX19	+	nd		H34
**BCCO 10_305**	*S. sp.*	-	nd	nd	BOX2	+	KP404464	colabomycin	H34
**BCCO 10_321**	*S. sp.*	-	nd	nd	BOX2	+	KP404596	colabomycin	H34
**BCCO 10_299**	*S. sp.*	-	nd	nd	BOX20	+	KP404463	colabomycin	H34
**BCCO 10_326**	*S. sanglieri (S. gelaticus)* SOK8/2-05	98.64	35	EU098042	BOX20	+	KP404597	colabomycin	H34
**BCCO 10_1122**[$]	*S. sp.*	-	nd	nd	BOX21	+	nd		H2
**BCCO 10_1123**[$]	*S. sp.*	-	nd	nd	BOX21	+	nd		H2
**BCCO 10_1666**	*S. hundungensis*	99.43	nd	KP718561	BOX22	+	KP404546	uknown - purple branch	H24
**BCCO 10_1669**	*S. hundungensis*	99.44	nd	KP718560	BOX22	+	KP404564	uknown - purple branch	H24
**BCCO 10_567**	*S. aureus*	98.59	26	KP718500	BOX23	+	KP404478	colabomycin	H37
**BCCO 10_859**	*S. sp.*	-	nd	nd	BOX23	negative	nd		H32
**BCCO 10_57**	*S. sp.*	-	nd	nd	BOX24	+	nd		H32
**BCCO 10_91**	*S. sp.*	-	nd	nd	BOX24	+	KP404451	moenomycin	H33
**BCCO 10_890**	*S. kanamyceticus*	98.79	nd	KP718564	BOX25	+	KP404580	colabomycin	H33
**BCCO 10_896**	*S. aureus*	99.00	26	KP718505	BOX25	+	KP404582	colabomycin	H33
**BCCO 10_1078**	*S. nojiriensis (S. levandulae)*	100.00	39	KP718579	BOX26	+	KP404563	ECO-02301	H15
**BCCO 10_1079**	*S. cavourensis (S. celluloflavus)*	99.93	nd	KP718519	BOX26	negative	nd		H15
**BCCO 10_300**	*S. sp.*	-	nd	nd	BOX27	+	nd		H34
**BCCO 10_988**	*S. sp.*	-	nd	nd	BOX27	+	nd		H50
**BCCO 10_524**	*S. scabiei*	99.72	25	KP718596	BOX28	+	KP404552	baf/moe cluster	H1
**BCCO 10_1254**	*S. sp.*	-	nd	nd	BOX28	+	nd		H3
**BCCO 10_1476**	*S. subrutilus (S. levandulae)*	99.91	39	KP718603	BOX28	+	KP404534	annimycin	H26
**BCCO 10_77**	*S. ederensis (S. umbrinus)*	100.00	18	KP718529	BOX29	+	KP404450	moenomycin	H32

**BCCO 10_863**	*S. phaeochromogenes*	100.00	18	KP718589	BOX29	+	KP404493	moenomycin	H32
**BCCO 10_10**	*S. sp.*	-	nd	nd	BOX3	negative	nd		H35
**BCCO 10_352**	*S. sp.*	-	nd	nd	BOX3	+	nd		H35
**BCCO 10_159**	*S. sp.*	-	nd	nd	BOX30	+	nd		H34
**BCCO 10_1356**	*S. phaeochromogenes*	100.00	18	KP718591	BOX30	+	KP404589	moenomycin	H52
**BCCO 10_112**	*S. sp.*	-	nd	nd	BOX31	+	nd		H32
**BCCO 10_242**	*S. sp.*	-	nd	nd	BOX31	+	KP404461	moenomycin	H33
**BCCO 10_48**	*S. sp.*	-	nd	nd	BOX32	+	KP404445	moenomycin	H32
**BCCO 10_53**	*S. aurantiacus*	99.50	19	KP718488	BOX32	+	KP404447	moenomycin	H32
**BCCO 10_59**	*S. sp.*	-	nd	nd	BOX32	+	KP404448	moenomycin	H32
**BCCO 10_628**	*S. ederensis (S. umbrinus)*	99.64	18	KP718535	BOX33	+	KP404588	moenomycin	H39
**BCCO 10_637**	*S. ederensis (S. umbrinus)*	99.79	18	KP718536	BOX33	+	KP404587	moenomycin	H34
**BCCO 10_393**	*S. ederensis (S. umbrinus)*	99.56	18	KP718531	BOX34	+	KP404470	moenomycin	H32
**BCCO 10_403**	*S. phaeochromogenes*	99.43	18	KP718587	BOX34	+	KP404472	moenomycin	H32
**BCCO 10_454**	*S. sp.*	-	nd	nd	BOX34	+	KP404474	moenomycin	H32
**BCCO 10_457**	*S. sp.*	-	nd	nd	BOX34	+	KP404560	moenomycin	H32
**BCCO 10_458**	*S. ederensis (S. umbrinus)*	99.93	18	KP718533	BOX34	+	nd		H32
**BCCO 10_394**	*S. aurantiacus*	99.51	19	KP718491	BOX35	+	KP404471	moenomycin	H32
**BCCO 10_887**	*S. sp.*	-	nd	nd	BOX35	+	KP404579	colabomycin	H33
**BCCO 10_1332**	*S. flavovirens (S. griseus)*	100.00	37	KP718540	BOX36	+	KP404526	blue, classical ALAS	H13
**BCCO 10_1341**	*S. prunicolor*	99.78	nd	KP718592	BOX36	+	nd		H13
**BCCO 10_1056**	*S. sp.*	-	nd	nd	BOX37	+	KP404512	annimycin	H48
**BCCO 10_1062**	*S. cirratus (S. levandulae)*	99.86	39	KP718523	BOX37	+	KP404514	annimycin	H48
**BCCO 10_39**	*S. sp.*	-	nd	nd	BOX4	negative	nd		H35
**BCCO 10_42**	*S. sp.*	-	nd	nd	BOX4	negative	nd		H35
**BCCO 10_191**	*S. griseoplanus (S. griseus)*	100.00	54	KP718547	BOX4	+	nd		H35
**BCCO 10_474**	*S. griseorubiginosus (S. griseus)*	99.58	7	KP718553	BOX5	+	KP404595	orange cluster	H36
**BCCO 10_1371**[&]	*S. laculatispora (S. brevispora)*	99.86	nd	KP718567	BOX5	+	KP404527	ECO-02301	H7
**BCCO 10_140**	*S. sp.*	-	nd	nd	BOX6	+	nd		H34
**BCCO 10_143**	*S. sp.*	-	nd	nd	BOX6	+	nd		H34
**BCCO 10_1400**	*S. sioyaensis*	99.93	nd	KP718600	BOX7	+	KP404530	annimycin	H17

**BCCO 10_1442**	*S. lincolnensis*	99.93	3	KP718569	BOX7	+	KP404532	moenomycin	H17
**BCCO 10_23**	*S. sp.*	-	nd	nd	BOX8	+	nd		H35
**BCCO 10_251**	*S. sp.*	-	nd	nd	BOX8	+	nd		H33
**BCCO 10_413**	*S. ederensis (S. umbrinus)*	100.00	18	KP718532	BOX9	+	nd		H32
**BCCO 10_1073**	*S. sp.*	-	nd	nd	BOX9	+	nd		H42
**BCCO 10_149**	*S. sp.*	-	nd	nd	nd	+	KP404454	colabomycin	H34
**BCCO 10_234**	*S. sp.*	-	nd	nd	nd	+	KP404559	moenomycin	H32
**BCCO 10_862**	*S. nojiriensis (S. levandulae)*	99.51	39	KP718574	nd	+	KP404492	moenomycin	H32
**BCCO 10_907**	*S. olivochromogenes*	98.72	20	KP718584	nd	+	KP404500	colabomycin	H33
**BCCO 10_1076**	*S. sp.*	-	nd	nd	nd	+	KP404562	ECO-02301	H15
**BCCO 10_1523**	*S. ederensis (S. umbrinus)*	100.00	18	KP718538	nd	+	KP404536	moenomycin	H27
**BCCO 10_1095**$^{\Delta}$	*S. bacillaris*	99.93	nd	KP718508	nd	+	KP404517	bafilomycin	H21
**K7/4***	*S. sp.*	-	nd	nd	nd	+	KP404553	moenomycin	H15
**BCCO 10_330**	*S. sp.*	-	nd	nd	unique	negative	nd		H34
**L170***	*S. carpaticus*	99.86	128	KP718518	unique	+	nd		H51
**BCCO 10_1**	*S. kanamyceticus*	99.36	nd	KP718562	unique	+	KP404444	colabomycin	H35
**BCCO 10_5**	*S. aureus* SOK1/5-04	98.80	26	EU098016	unique	+	KP404586	colabomycin	H35
**BCCO 10_26**	*S. sp.*	-	nd	nd	unique	+	nd		H35
**BCCO 10_52**	*S. novaecaesareae*	99.65	nd	KP718580	unique	+	KP404446	blue, classical ALAS	H32
**BCCO 10_74**	*S. sp.*	-	nd	nd	unique	+	nd		H32
**BCCO 10_84**	*S. sp.*	-	nd	nd	unique	+	nd		H32
**BCCO 10_95**	*S. sp.*	-	nd	nd	unique	+	nd		H33
**BCCO 10_106**	*S. sp.*	-	nd	nd	unique	+	nd		H33
**BCCO 10_120**	*S. sp.*	-	nd	nd	unique	+	nd		H33
**BCCO 10_127**	*S. sp.*	-	nd	nd	unique	+	nd		H34
**BCCO 10_129**	*S. sp.*	-	nd	nd	unique	+	KP404453	blue, classical ALAS	H34
**BCCO 10_145**	*S. sp.*	-	nd	nd	unique	+	nd		H34
**BCCO 10_147**	*S. sp.*	-	nd	nd	unique	+	nd		H34
**BCCO 10_148**	*S. sp.*	-	nd	nd	unique	+	nd		H34
**BCCO 10_157**	*S. sp.*	-	nd	nd	unique	+	nd		H34
**BCCO 10_161**	*S. aureus*	98.93	26	KP718497	unique	+	nd		H34

**BCCO 10_163**	*S. sp.*	-	nd	nd	unique	+	nd		H34
**BCCO 10_166**	*S. sp.*	-	nd	nd	unique	+	nd		H34
**BCCO 10_173**	*S. sp.*	-	nd	nd	unique	+	nd		H35
**BCCO 10_175**	*S. griseoplanus (S. griseus)*	100.00	54	KP718546	unique	+	KP404455	annimycin	H35
**BCCO 10_189**	*S. sp.*	-	nd	nd	unique	+	nd		H35
**BCCO 10_194**	*S. sp.*	-	nd	nd	unique	+	nd		H35
**BCCO 10_202**	*S. sp.*	-	nd	nd	unique	+	nd		H32
**BCCO 10_203**	*S. sp.*	-	nd	nd	unique	+	KP404456	moenomycin	H32
**BCCO 10_210**	*S. sp.*	-	nd	nd	unique	+	KP404458	blue, classical ALAS	H32
**BCCO 10_215**	*S. graminilatus*	98.94	nd	KP718543	unique	+	KP404459	manumycin	H32
**BCCO 10_217**	*S. sp.*	-	nd	nd	unique	+	nd		H32
**BCCO 10_237**	*Amycolatopsis thailandensis*	99.71	nd	KP718483	unique	+	KP404460	reductiomycin	H32
**BCCO 10_238**	*S. sp.*	-	nd	nd	unique	+	nd		H32
**BCCO 10_248**	*S. sp.*	-	nd	nd	unique	negative	nd		H33
**BCCO 10_250**	*S. yanii (S. gelaticus)* SOK5/7-05	100.00	35	EU098027	unique	+	nd		H33
**BCCO 10_272**	*S. phaeochromogenes* SOK6/4-05	99.79	18	EU098032	unique	negative	nd		H33
**BCCO 10_302**	*S. olivochromogenes* SOK7/8-05	98.72	20	EU098037	unique	+	KP404569	colabomycin	H34
**BCCO 10_311**	*Kitasatospora gansuensis* SOK7/17-05	99.72	59	EU098040	unique	+	nd		H34
**BCCO 10_313**	*S. kanamyceticus*	99.35	nd	KP718563	unique	+	KP404567	colabomycin	H34
**BCCO 10_314**	*S. sp.*	-	nd	nd	unique	+	KP404465	colabomycin	H34
**BCCO 10_317**	*S. mauvecolor (S. somaliensis)*	100.00	29	KP718570	unique	+	KP404466	blue, classical ALAS	H34
**BCCO 10_333**	*S. sp.*	-	nd	nd	unique	+	KP404468	colabomycin	H34
**BCCO 10_334**	*S. sp.*	-	nd	nd	unique	+	KP404572	colabomycin	H34
**BCCO 10_336**	*S. sp.*	-	nd	nd	unique	+	KP404584	colabomycin	H34
**BCCO 10_343**	*S. sp.*	-	nd	nd	unique	+	KP404575	colabomycin	H34
**BCCO 10_373**	*S. sp.*	-	nd	nd	unique	+	KP404469	blue, classical ALAS	H35
**BCCO 10_398**	*S. aurantiacus*	99.51	19	KP718492	unique	+	KP404556	moenomycin	H32
**BCCO 10_406**	*S. aurantiacus*	99.27	19	KP718493	unique	+	KP404473	moenomycin	H32
**BCCO 10_419**	*S. sp.*	-	nd	nd	unique	+	nd		H32
**BCCO 10_495**	*S. sp.*	-	nd	nd	unique	+	KP404475	bafilomycin	H14

**BCCO 10_534**	*S. sp.*	-	nd	nd	unique	+	nd		H9
**BCCO 10_544**	*S. griseoplanus (S. griseus)*	100.00	54	KP718548	unique	+	KP404476	colabomycin	H8
**BCCO 10_565**	*S. aurantiacus*	99.37	19	KP718494	unique	+	KP404551	moenomycin	H35
**BCCO 10_570**	*S. sp.*	-	nd	nd	unique	negative	nd		H43
**BCCO 10_592**	*S. sp.*	-	nd	nd	unique	negative	nd		H34
**BCCO 10_607**	*S. ederensis (S. umbrinus)*	100.00	18	KP718534	unique	+	KP404550	moenomycin	H38
**BCCO 10_623**	*S. aureus*	98.94	26	KP718501	unique	+	KP404480	colabomycin	H39
**BCCO 10_631**	*S. sp.*	-	nd	nd	unique	+	nd		H32
**BCCO 10_639**	*S. ederensis (S. umbrinus)*	99.93	18	KP718537	unique	+	KP404481	moenomycin	H38
**BCCO 10_650**	*S. sp.*	-	nd	nd	unique	+	KP404482	orange cluster	H41
**BCCO 10_661**	*S. aureus*	98.88	26	KP718502	unique	+	KP404549	colabomycin	H40
**BCCO 10_666**	*S. griseoplanus (S. griseus)*	100.00	nd	KP718487	unique	+	KP404484	orange cluster	H5
**BCCO 10_670**	*S. griseoplanus (S. griseus)*	100.00	54	KP718549	unique	+	KP404565	orange cluster	H5
**BCCO 10_708**	*S. sp.*	-	nd	nd	unique	+	nd		H4
**BCCO 10_740**	*S. finlayi (S. clavifer)*	100.00	34	KP718539	unique	+	KP404485	blue, classical ALAS	H6
**BCCO 10_741**	*S. sp.*	-	nd	nd	unique	+	KP404486	blue, classical ALAS	H6
**BCCO 10_790**	*Lentzea violacea*	99.43	nd	KP718485	unique	+	KP404488	annimycin	H35
**BCCO 10_806**	*Saccharothrix espanaensis*	99.63	nd	KP718605	unique	+	KP404490	brown cluster	H33
**BCCO 10_877**	*S. nojiriensis (S. levandulae)*	100.00	39	KP718575	unique	+	KP404494	blue, classical ALAS	H33
**BCCO 10_878**	*S. nojiriensis (S. levandulae)*	100.00	39	KP718576	unique	+	KP404495	blue, classical ALAS	H33
**BCCO 10_883**	*S. sp.*	-	nd	nd	unique	negative	nd		H33
**BCCO 10_892**	*S. sp.*	-	nd	nd	unique	+	KP404498	colabomycin	H33
**BCCO 10_895**	*S. sp.*	-	nd	nd	unique	+	KP404581	colabomycin	H33
**BCCO 10_900**	*S. kanamyceticus*	98.84	nd	KP718566	unique	+	KP404499	colabomycin	H33
**BCCO 10_905**	*S. sp.*	-	nd	nd	unique	negative	nd		H33
**BCCO 10_912**	*S. sp.*	-	nd	nd	unique	+	KP404501	colabomycin	H33
**BCCO 10_916**	*S. sp.*	-	nd	nd	unique	+	nd		H34
**BCCO 10_918**	*S. caeruleatus*	99.71	3	KP718511	unique	+	KP404504	orange cluster	H34
**BCCO 10_923**	*S. sp.*	-	nd	nd	unique	+	KP404505	moenomycin	H47
**BCCO 10_930**	*S. celluloflavus*	99.57	31	KP718521	unique	+	KP404506	orange cluster	H46
**BCCO 10_947**	*S. caeruleatus*	99.71	3	KP718512	unique	+	KP404590	moenomycin	H47

**BCCO 10_948**	*Saccharothrix espanaensis*	99.65	nd	KP718606	unique	+	KP404510	brown cluster	H46
**BCCO 10_981**	*S. sioyaensis*	99.84	nd	KP718598	unique	+	KP404593	annimycin	H49
**BCCO 10_1021**	*S. griseoplanus (S. griseus)*	99.93	54	KP718550	unique	+	KP404511	annimycin	H53
**BCCO 10_1038**	*S. yanii (S. gelaticus)*	100.00	35	KP718604	unique	+	nd		H48
**BCCO 10_1059**	*S. nojiriensis (S. levandulae)*	100.00	39	KP718577	unique	+	KP404513	ECO-02301	H48
**BCCO 10_1065**	*S. sp.*	-	nd	nd	unique	+	KP404515	annimycin	H48
**BCCO 10_1077**	*S. sp.*	-	nd	nd	unique	+	nd		H16
**BCCO 10_1222**	*S. galilaeus (S. bobili)*	100.00	13	KP718542	unique	+	KP404522	annimycin	H3
**BCCO 10_1229**	*S. sp.*	-	nd	nd	unique	+	KP404523	blue, classical ALAS	H3
**BCCO 10_1270**	*S. griseorubens (S. griseoflavus)*	99.79	100	KP718552	unique	+	KP404525	annimycin	H3
**BCCO 10_1336**	*S. canus*	99.86	8	KP718516	unique	+	nd		H13
**BCCO 10_1344**	*S. sioyaensis*	99.57	nd	KP718599	unique	+	nd		H13
**BCCO 10_1394**	*S. calvus*	100.00	118	KP718514	unique	+	KP404529	annimycin	H17
**BCCO 10_1405**	*S. sioyaensis*	99.86	nd	KP718601	unique	+	KP404531	baf/moe cluster	H17
**BCCO 10_1440**	*S. calvus*	100.00	118	KP718515	unique	+	KP404561	annimycin	H17
**BCCO 10_1467**	*S. aureus*	99.93	26	KP718506	unique	+	nd		H30
**BCCO 10_1471**	*S. griseoflavus*	99.79	nd	KP718544	unique	+	KP404533	bafilomycin	H29
**BCCO 10_1496**	*S. scabisporus*	100.00	nd	KP718597	unique	+	nd		H27
**BCCO 10_1514**	*S. cyaneofuscatus (S. griseus)*	100.00	nd	KP718526	unique	+	KP404535	blue, classical ALAS	H26
**BCCO 10_1524**	*S. sp.*	-	nd	nd	unique	negative	nd		H28
**BCCO 10_1548**	*S. curacoi*	99.44	nd	KP718525	unique	+	KP404537	orange cluster	H44
**BCCO 10_1549**	*S. caeruleatus*	99.57	3	KP718513	unique	+	KP404538	moenomycin	H44
**BCCO 10_1582**	*S. sp.*	-	nd	nd	unique	+	KP404540	blue, classical ALAS	H17
**BCCO 10_1633**	*Kitasatospora atroaurantiaca*	99.58	59	KP718484	unique	+	KP404541	annimycin	H22
**BCCO 10_1636**	*S. capoamus*	98.88	nd	KP718517	unique	+	KP404542	manumycin	H22
**BCCO 10_1649**	*S. galbus*	99.37	1	KP718541	unique	+	KP404543	annimycin	H22
**BCCO 10_1658**	*Nocardia nova*	97.65	nd	KP718486	unique	+	KP404544	blue, classical ALAS	H23
**BCCO 10_1664**	*S. clavuligerus*	99.72	nd	KP718524	unique	+	KP404545	moenomycin	H22
**BCCO 10_1671**	*S. mirabilis*	99.72	20	KP718572	unique	+	KP404547	annimycin	H24
**BCCO 10_1093**$^{\Delta}$	*S. cavourensis (S. celluloflavus)*	99.93	nd	KP718520	unique	+	nd		H20

**BCCO 10_1094**[Δ]	*S. sp.*	-	nd	nd	unique	+	KP404516	bafilomycin	H20
**BCCO 10_1096**[Δ]	*S. sp.*	-	nd	nd	unique	+	KP404518	bafilomycin	H21
**BCCO 10_1100**[Δ]	*S. bacillaris*	99.93	nd	KP718509	unique	+	KP404591	bafilomycin	H19
**BCCO 10_1110**[$]	*S. sp.*	-	nd	nd	unique	+	nd		H2
**BCCO 10_1121**[$]	*S. sp.*	-	nd	nd	unique	+	nd		H2
**BCCO 10_1124**[$]	*S. lienomycini (S. tendae)*	99.44	103	KP718568	unique	+	KP404594	bafilomycin	H2
**BCCO 10_1127**[$]	*S. sp.*	-	nd	nd	unique	+	KP404585	colabomycin	H2
**BCCO 10_1190**[$]	*S. griseoplanus (S. griseus)*	100.00	54	KP718551	unique	+	KP404521	blue, classical ALAS	H2
**BCCO 10_1375**[&]	*S. griseolus (S. griseus)*	100.00	36	KP718545	unique	+	KP404528	bafilomycin	H11
**BCCO 10_769**[#]	*S. mauvecolor (S. somaliensis)*	100.00	29	KP718571	unique	+	KP404487	blue, classical ALAS	H10
**L037***	*S. cyaneofuscatus (S. griseus)*	100.00	nd	KP718527	unique	+	nd		H51
**W24-08***	*S. sioyaensis*	99.72	nd	KP718602	unique	+	KP404592	annimycin	H50
**BCCO 10_56**	*S. aurantiacus*	99.50	19	KP718489	unique	+	nd		H32
**BCCO 10_57**	*S. sp.*	-	nd	nd	unique	+	nd		H32
**BCCO 10_75**	*S. ederensis (S. umbrinus)*	100.00	18	KP718528	unique	+	KP404449	moenomycin	H32
**BCCO 10_107**	*S. sp.*	-	nd	nd	unique	+	KP404452	orange cluster	H33
**BCCO 10_158**	*S. sp.*	-	nd	nd	unique	+	nd		H34
**BCCO 10_212**	*S. aurantiacus*	99.50	19	KP718490	unique	+	KP404566	moenomycin	H32
**BCCO 10_322**	*S. avidinii (S. lavendulae)*	99.72	39	KP718507	unique	+	KP404467	uknown - purple branch	H34
**BCCO 10_338**	*S. sp.*	-	nd	nd	unique	+	KP404574	colabomycin	H34
**BCCO 10_558**	*S. sp.*	-	nd	nd	unique	+	KP404555	orange cluster	H31
**BCCO 10_566**	*S. aurantiacus*	99.51	19	KP718495	unique	+	KP404477	moenomycin	H35
**BCCO 10_590**	*S. sp.*	-	nd	nd	unique	negative	nd		H34
**BCCO 10_649**	*S. sp.*	-	nd	nd	unique	+	nd		H41
**BCCO 10_659**	*S. sp.*	-	nd	nd	unique	+	KP404483	colabomycin	H40
**BCCO 10_799**	*S. aurantiacus*	99.51	19	KP718496	unique	+	KP404489	moenomycin	H32
**BCCO 10_857**	*S. phaeochromogenes*	100.00	18	KP718588	unique	+	KP404491	moenomycin	H32
**BCCO 10_880**	*S. aureus*	99.07	nd	KP718504	unique	+	KP404496	blue, classical ALAS	H33
**BCCO 10_934**	*S. purpeofuscus*	100.00	nd	KP718593	unique	+	KP404507	bafilomycin	H46

**BCCO 10_936**	*S. purpeofuscus*	100.00	nd	KP718594	unique	+	KP404508	bafilomycin	H46
**BCCO 10_942**	*S. griseus subsp. griseus (S. griseus)*	99.58	112	KP718558	unique	+	KP404509	blue, classical ALAS	H45
**BCCO 10_1074**	*S. nojiriensis (S. levandulae)*	100.00	39	KP718578	unique	+	nd		H15
**BCCO 10_1218**	*S. sp.*	-	nd	nd	unique	+	nd		H3
**BCCO 10_1247**	*S. sp.*	-	nd	nd	unique	+	KP404524	blue, classical ALAS	H3
**BCCO 10_1552**	*S. monomycini*	99.51	73	KP718573	unique	+	KP404539	uknown - purple branch	H44
**BCCO 10_1767**	*S. rubrogriseus (S. tendae)*	98.31	103	KP718595	unique	+	KP404548	bafilomycin	H25
**BCCO 10_1097**[Δ]	*S. phaeochromogenes*	99.44	18	KP718590	unique	+	KP404519	bafilomycin	H21
**BCCO 10_1104**[Δ]	*S. celluloflavus*	99.55	31	KP718522	unique	+	KP404520	bafilomycin	H18

* Strains not included in the BCCO (www.actinomycetes.cz)
** Phylogenetic clades were assigned according to Labeda *et al.*, 2012.
[#] Strain isolated and provided by Soňa Javoreková, Slovak University of Agriculture in Nitra, Slovakia
[&]Strains isolated and provided by Dana Elhottová, Biology Centre CAS, v. v. i., České Budějovice, Czech Republic
[$] Strains isolated and provided by Mika Tarkka, Helmholtz Centre for Environmental Research, Halle, Germany
[Δ] Strains isolated and provided by Miroslav Kolařík, Institute of Microbiology CAS, v. v. i., Prague, Czech Republic

Reference

Labeda, D.P., Goodfellow, M., Brown, R., Ward, A.C., Lanoot, B., Vanncanneyt , M., Swings, J., Kim, S.B., Liu, Z., Chun, J., Tamura, T., Oguchi, A., Kikuchi, T., Kikuchi, H., Nishii, T., Tsuji, K., Yamaguchi, Y., Tase, A., Takahashi, M., Sakane, T., Suzuki, K.I., Hatano, K. (2012). Phylogenetic study of the species within the family *Streptomycetaceae*. Antonie *Van Leeuwenhoek* **101**, 73-104.

Supplementary Table 2. Description of habitats as sources of environmental Streptomycetes strains. The codes, isolation sources, localities, and references are listed. Moreover, the detection and phylogenetic relatedness of *hemA* gene sequence in studied habitat is given.

Habitat code	Habitat/Isolation source	Locality, country	*hemA* clusters	Reference
**H1**	soil above a cave	Ardovská Cave, Slovakia	baf/moe	
**H2**	agricultural soil, cellulose enrichment	Bad Lauchstädt, Germany	bafilomycin, colabomycin, blue	Gutknecht *et al*., 2012
**H3**	agricultural soil, chitin enrichment	Bad Lauchstädt, Germany	linear polyketides, blue	Gutknecht *et al*., 2012
**H4**	winter pasture, soil with moderate cattle impact	Borová farm, Czech Republic	*nd*	Chroňáková *et al*., 2009b
**H5**	winter pasture, soil with severe cattle impact	Borová farm, Czech Republic	orange	Chroňáková *et al*., 2009b
**H6**	winter pasture, soil without cattle impact	Borová farm, Czech Republic	blue	Chroňáková *et al*., 2009b
**H7**	bat guano	Domica Cave, Slovakia	linear polyketides	Kyselková *et al*., 2012
**H8**	river sediment	Domica Cave, Slovakia	colabomycin	
**H9**	soil above a cave	Domica Cave, Slovakia	*nd*	
**H10**	mountain soil	Donovaly (Low Tatra Mts.), Slovakia	blue	
**H11**	bat guano	Drieňovská Cave, Slovakia	bafilomycin	
**H12**	forefield of glacier	Grindelwald glacier, Switzerland	*nd*	
**H13**	forest soil	Holubov, Czech Republic	blue	
**H14**	soil near old beehive	Chelčice, Czech Republic	bafilomycin	
**H15**	meadow, manured soil	Chmelná, Czech Republic	linear polyketides, moenomycin	Kyselková *et al*., 2012
**H16**	recultivation area, soil	Illinois, USA	*nd*	Frouz *et al*., 2013
**H17**	technozem	Kryvyi Rig, Ukraine	baf/moe, linear polyketides, moenomycin, blue	
**H18**	beetle gallery (*Crossotarsus mniszechi*)	Madang prov., Utai village, Papua New Guinea	bafilomycin	Hulcr and Cognato, 2010
**H19**	beetle gallery (*Diapus pussilimus*)	Madang prov., Utai village, Papua New Guinea	bafilomycin	Hulcr and Cognato, 2010

**H20**	beetle gallery (*Dinoplatypus pallidus*)	Madang prov., Utai village, Papua New Guinea	bafilomycin	Hulcr and Cognato, 2010
**H21**	beetle gallery (*Xyleborus perforans*)	Madang prov., Utai village, Papua New Guinea	bafilomycin	Hulcr and Cognato, 2010
**H22**	foggy forest, soil	Mount Cameroon, Cameroon	linear polyketides, moenomycin, manumycin	
**H23**	lowland forest, soil	Mount Cameroon, Cameroon	blue	
**H24**	savanna soil	Mount Cameroon, Cameroon	linear polyketides, unknown	
**H25**	soil near old beehive	Nové Hrady, Czech Republic	bafilomycin	
**H26**	pathway sediment, entrance to the cave	Planinska jama, Slovenia	linear polyketides, blue	
**H27**	forest soil above a cave	Planinska jama, Slovenia	moenomycins	
**H28**	river sediment outside the cave	Planinska jama, Slovenia	*nd*	
**H29**	meadow river bed, soil	Rakov Škocjan, Slovenia	bafilomycin	
**H30**	forest soil	Rakov Škocjan, Slovenia	*nd*	
**H31**	Miocene sediment, 200 m below the surface	Sokolov brown coal mining area, Czech Republic	orange	Chroňáková *et al.,* 2010
**H32**	soil, 10 y. old spontaneous succession	Sokolov brown coal mining area, Czech Republic	moenomycin, manumycin, reductiomycin, blue	Chroňáková *et al.,* 2010
**H33**	soil, 20 y. old spontaneous succession	Sokolov brown coal mining area, Czech Republic	moenomycin, colabomycin, orange, brown, blue	Chroňáková *et al.,* 2010
**H34**	soil, climax, spontaneous succession	Sokolov brown coal mining area, Czech Republic	moenomycin, colabomycin, orange, unknown, blue	Chroňáková *et al.,* 2010
**H35**	initial stage of spontaneous succession	Sokolov brown coal mining area, Czech Republic	linear polyketides, moenomycin, colabomycin, blue	Chroňáková *et al.,* 2010
**H36**	soil, microcosm experiment	Sokolov brown coal mining area, Czech Republic	orange	
**H37**	soil vegetated by aspen and sallow	Sokolov brown coal mining area, Czech Republic	colabomycin	
**H38**	soil vegetated by birch	Sokolov brown coal mining area, Czech Republic	moenomycins	
**H39**	soil vegetated by birch, alder and larch	Sokolov brown coal mining area, Czech Republic	colabomycin, moenomycin	
**H40**	soil vegetated by birch, aspen	Sokolov brown coal mining area, Czech Republic	colabomycin	
**H41**	soil vegetated by birch, sallow and herb	Sokolov brown coal mining area, Czech Republic	orange	
**H42**	soil vegetated by sallow	Sokolov brown coal mining area, Czech Republic	*nd*	

**H43**	soil vegetated by sallow and aspen	Sokolov brown coal mining area, Czech Republic	orange	
**H44**	botanical garden, sandy soil	Tbilisi, Georgia	moenomycin, orange, unknown	
**H45**	10-15 years old spontaneous succession	Tennesee, USA	blue	Frouz *et al*., 2013
**H46**	20-25 years old recultivation, soil	Tennesee, USA	bafilomycin, brown, orange	Frouz *et al*., 2013
**H47**	5 years old recultivation, soil	Tennesee, USA	moenomycin	Frouz *et al*., 2013
**H48**	soil from potatoes field	Vyklantice, Czech Republic	linear polyketides	
**H49**	6 years old recultivation, soil	Wyoming, USA	linear polyketides	Frouz *et al*., 2013
**H50**	native prairie, soil vegetated by sedge bush, opuncia	Wyoming, USA	linear polyketides	Frouz *et al*., 2013
**H51**	marine sediment	Yellow sea, China	bafilomycin, colabomycin, blue	
**H52**	soil from potatoes field	Ždírec, Czech Republic	moenomycin	
**H53**	soil from potatoes field	Židovice, Czech Republic	linear polyketides	

References

Chroňáková, A., Horák, A., Elhottová, D., Krištůfek, V. (2009a). Diverse archaeal community of a bat guano pile in Domica Cave (Slovak Karst, Slovakia). *Folia Microbiol* .**54**,436-446.

Chroňáková, A., Krištůfek, V., Tichý, M., Elhottová, D. (2010). Biodiversity of streptomycetes isolated from a succession sequence at a post-mining site and their evidence in Miocene lacustrine sediment. *Microbiol. Res.* **165**, 594-608.

Chroňáková, A., Rádl, V., Čuhel, J., Šimek, M., Elhottová, D., Engel, M., Schloter, M. (2009b). Overwintering management on upland pasture causes shifts in an abundance of denitrifying microbial communities, their activity and N_2O-reducing ability. *Soil Biol. Biochem.* **41**, 1132-1138.

Frouz, J., Jilková, V., Cajthaml, T., Pižl, V., Tajovský, K., Hanel, L., *et al.* (2013). Soil biota in post-mining sites along a climatic gradient in the USA: Simple communities in shortgrass prairie recover faster than complex communities in tallgrass prairie and forest. *Soil Biol. Biochem*. **67**, 212-225.

Gutknecht, J., Schulz, E., Vemula, V., Schmidt, J., Henning, G., Rose, J., *et al.* (2012). Changes in enzyme activities and in the functional diversity of actinomycetes due to long term agricultural management. *Nature Precedings* doi: 10.1038/npre.2012.6792.

Hulcr, J., and Cognato, A.I. (2010). Repeated evolution of crop theft in Fungus-farming ambrosia beetles. *Evolution* **64**, 3205-3212.

Kyselková, M., Chroňáková, A., Volná, L., Němec, J., Ulmann, V., Scharfen, J., Elhottová, D. (2012). Tetracycline resistance and presence of tetracycline resistance determinants tet(V) and tap in rapidly growing mycobacteria from agricultural soils and clinical isolates. *Microbes Environ* **27**, 413-422.

Supplementary Table 3. List of reference ALAS proteins and genome-scanning fits with Accession Nos.

ALAS type	Organism, locus identifier (if needed)	ALAS Acc. No.	Compounds	Metabolite/pathway reference
CLASSICAL ALAS	***Aspergillus nidulans R153***	CAA45508		
	***Chromobacterium violaceum* ATCC12472**	WP_011134358		
	Homo sapiens	CAA39794		
	Mus musculus	NP_033783		
	***Nocardia brasiliensis* ATCC700358**	AFU00815		
	***Pseudomonas aeruginosa* HB15**	ESQ65936		
	***Rhodobacter capsulatus* SB1003**	YP_003577599		
	***Schizosaccharomyces pombe* 972h-**	CAB16265		
CYCLISING ALAS, TYPE PRODUCER STRAINS OF C_5N COMPOUNDS	***Kitasatospora cheerisanensis* KTCC2395**	YP_004909054	**bafilomycins**	Hwang *et al*., 2015
	***Kitasatospora setae* KM-6054**	KDN87766	**bafilomycins**	Ichikawa *et al*., 2010
	***Streptomyces aizunensis* NRRL B-11277**	AAX98209	**ECO-02301**	McAlpine *et al*., 2005
	***S. aureus* SOK1/5-04**	AIL50190	**colabomycins**	Petříčková *et al*., 2014
	***S. bambergiensis* ATCC13879**	KP283530	**moenomycins**	Ostash *et al*., 2010, This work
	***S. calvus* ATCC13382**	AGY30678	**annimycin**	Kalan *et al*., 2013
	***S. clavuligerus* ATCC27064**	WP_003963595	**moenomycins**	Ostash *et al*., 2010
	S. ghanaensis* ATCC14672, *moeA5	ABJ90153	**moenomycins**	Ostash *et al*., 2007
	S. ghanaensis* ATCC14672, *moeC4	ABJ90148	**moenomycins**	Ostash *et al*., 2007
	***S. griseoaurantiacus* M045**	EGG47995	**manumycins, chinikomycins**	Li *et al*., 2005
	***S. griseus* DSM2608**	AGK25193	**bafilomycins**	Hwang *et al*., 2013
	***S. hygroscopicus* ATCC31955**	CCF23198	**L-155,175 bafilomycin-type**	Kim *et al*., 2012
	***S. lohii* ATCC BAA-1276**	ADC79615	**bafilomycins**	Zhang *et al*., 2013
	***S. nodosus ssp. asukaensis* ATCC29757**	AAO62615	**asukamycin, manumycins**	Rui et al., 2010
	***S. parvulus* Tü64**	KP283531	**manumycins**	Zeeck *et al*., 1987, This work
	S. xanthochromogenes	KP283529	**reductiomycin**	Shimizu *et al*., 1981, This work
GENOME SCANNING FITS	***Amycolatopsis orientalis* HCCB10007**	YP_008011931		
	***S. griseus griseus* NBRC13350**	YP_001826807		
	***S. peruviensis* NRRL Isp.-5592**	WP_030051431		
	***S. peuceticus* NRRL WC-3868**	WP_031191678		
	***S. rapamycinicus* NRLL5491**	YP_008795594		
	***S. resistomycificus* NRRL Isp.-5133**	WP_030044707		
	***S. rimosus rimosus* ATCC10970**	ELQ83458		
	***S. roseochromogenes oscitans* DS12.976,**	EST29037		
	***S. roseochromogenes oscitans* DS12.976,**	EST29040		
	***S. seoulensis* NRRL B-24310**	WP_031183405		
	***S. sp.* 351MFTsu5.1**	WP_020139869		
	***S. sp.* CcalMP-8W**	WP_018487281		
	***S. sp.* CNB091**	WP_018955923		
	***S. sp.* DPondAA-B6**	WP_028440470		
	***S. sp.* NRRL F-2305**	WP_030834459		
	***S. sp.* NRRL WC-3742**	WP_031065778		

***S. sp.* NTK 937**	KDQ71152
***S. sp.* PCS3-D2**	EYU71398
***S. sp.* PRh5**	EXU64037
***S. sp.* PsTaAH-124**	WP_018566618
***S. sp.* SirexAA-E**	AEN10069
***S. sp.* XylebKG-1, SACT1_0214**	EGE39623
***S. sp.* XylebKG-1, SACT1_5610**	EGE44919
***S. sp.* SolWspMP-sol2th**	WP_028419797
***S. sp.* Tü6176**	EYT82310
***S. viridosporus* T7A, STRVIR_RS0100400**	WP_016823064
***S. viridosporus* T7A, STRVIR_RS0105800**	WP_016823888
***Streptosporangium roseum* DSM43021**	ACZ91854
***Saccharothrix espanaensis* DSM44229,**	YP_007036324
***Saccharothrix espanaensis* DSM44229,**	YP_007038998
***Saccharothrix espanaensis* DSM44229,**	YP_007039164

References

Hwang, J.Y., Kim, H.S., Kim, S.H., Oh, H.R., Nam, D.H. (2013). Organization and characterization of a biosynthetic gene cluster for bafilomycin from *Streptomyces griseus* DSM 2608. *AMB Express* **3**, 24.

Hwang, J.Y., Kim, S.H., Oh, H.R., Kwon, E., Nam, D.H. (2015). Analysis of a draft genome sequence of *Kitasatospora cheerisanensis* KCTC 2395 producing bafilomycin antibiotics. *J. Microbiol.* **53**, 84-89.

Ichikawa, N., Oguchi, A., Ikeda, H., Ishikawa, J., Kitani, S., Watanabe, Y., *et al.* (2010). Genome Sequence of *Kitasatospora setae* NBRC 14216(T): An Evolutionary Snapshot of the Family *Streptomycetacea*e. *DNA Res.* **17**,393-406.

Kalan, L., Gessner, A., Thaker, M.N., Waglechner, N., Zhu, X.M., Szawiola, A., *et al.* (2013). A cryptic polyene biosynthetic gene cluster in *Streptomyces calvus* is expressed upon complementation with a functional *bldA* Gene. *Chem. Biol.* **20**, 1214-1224.

Kim, E.Y., Han, J.W., Lee, J.Y., Kim, B.S. (2012). Identification of the biosynthetic gene cluster for the antibiotic polyketide L-155,175 in *Streptomyces hygroscopicus*. *Folia Microbiol.* **57**, 543-550.

Li, F., Maskey, R.P., Qin, S., Sattler, I., Fiebig, H.H., Maier, A., *et al.* (2005). Chinikomycins A and B: isolation, structure elucidation, and biological activity of novel antibiotics from a marine Streptomyces sp. isolate M045. *J. Nat. Prod.* **68**, 349-353.

McAlpine, J.B., Bachmann, B.O., Piraee, M., Tremblay, S., Alarco, A.M., Zazopoulos, E., Farnet, C.M. (2005). Microbial Genomics as a guide to drug discovery and structural elucidation: ECO-02301, a novel antifungal agent, as an example. *J. Nat. Prod.* **68**, 493-496.

Ostash, B., Saghatelian, A., Walker, S. (2007). A streamlined metabolic pathway for the biosynthesis of moenomycin A. *Chem. Biol.* **14**, 257-267.

Ostash, B., and Walker, S. (2010). Moenomycin family antibiotics: chemical synthesis, biosynthesis, and biological activity. *Nat. Prod. Rep.* **27**, 1594-1617.

Petříčková, K., Pospíšil, S., Kuzma, M., Tylová, T., Jágr, M., Tomek, P., *et al.* (2014). Biosynthesis of colabomycin E, a new manumycin-family metabolite, involves an unusual chain-length factor. *Chembiochem* **15**, 1334-1345.

Rui, Z., Petříčková, K., Škanta, F., Pospíšil, S., Yang, Y.L., Chen, C.Y., *et al.* (2010). Biochemical and genetic insights into asukamycin biosynthesis. *J. Biol. Chem.* **285**, 24915-24924.

Shimizu, K., and Tamura, G. (1981). Reductiomycin, a new antibiotic .1. Taxonomy, fermentation, isolation, characterization and biological activities. *J. Antibiot.* **34**, 649-653.

Zeeck, A., Schroder, K., Frobel, K., Grote, R., Thiericke, R. (1987). The structure of manumycin .1. Characterization, structure elucidation and biological activity. *J. Antibiot*. **40**, 1530-1540.

Zhang, W., Fortman, J.L., Carlson, J.C., Yan, J.Y., Liu, Y., Bai, F.L., *et al.* (2013). Characterization of the bafilomycin biosynthetic gene cluster from *Streptomyces lohii*. *ChemBioChem* **14**, 301-306.

Supplementary Figure 1. 5-ALA biosynthetic pathways. 5-ALA, as the key precursor of tetrapyrrole compounds, is synthesized by two different pathways in different organisms: C5 pathway operates in most bacteria and plants, C4 in animals, fungi and proteobacteria. In rare cases, one organism uses both pathways in a single cell. As an example, some actinomycetes evolved a novel form of the key C4 pathway 5-ALA syntesizing enzyme, cyclizing aminolevulinate synthase (cALAS), which is able to cyclize the nascent 5-ALA and form the 2-amino-3-hydroxy-cyclopent-2-enone, the C_5N unit. The unit is then attached to various bioactive secondary metabolites. In this case, the C5 pathway supplements solely primary metabolism demands, whereas the C4 pathway provides precursors for secondary metabolism. Adapted from Petricek *et al.*, 2006 (*J. Bacteriol.* **188**, 5113-5123).

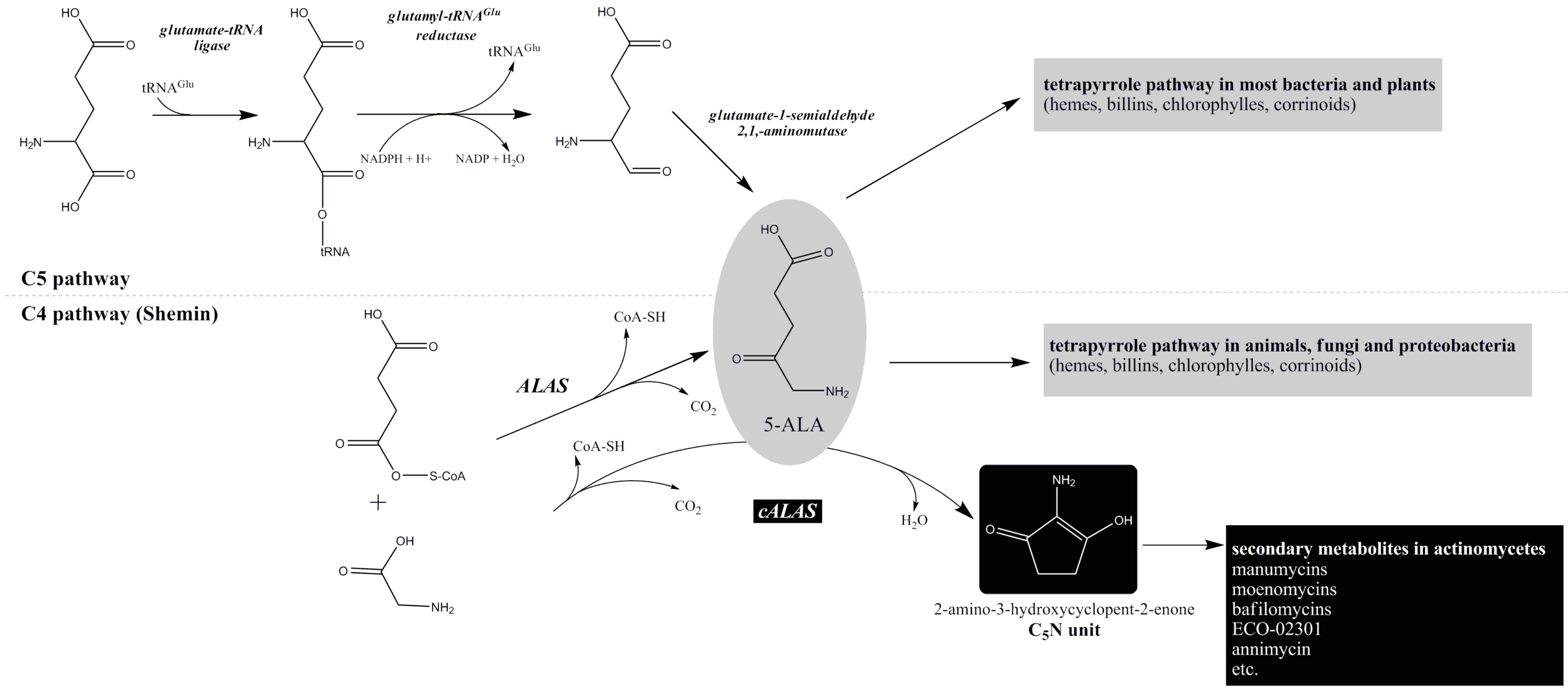

Supplementary Figure 2. Overview of actinomycete metabolites with C_5N unit and their major biological activities. The C_5N units are shown in blue.

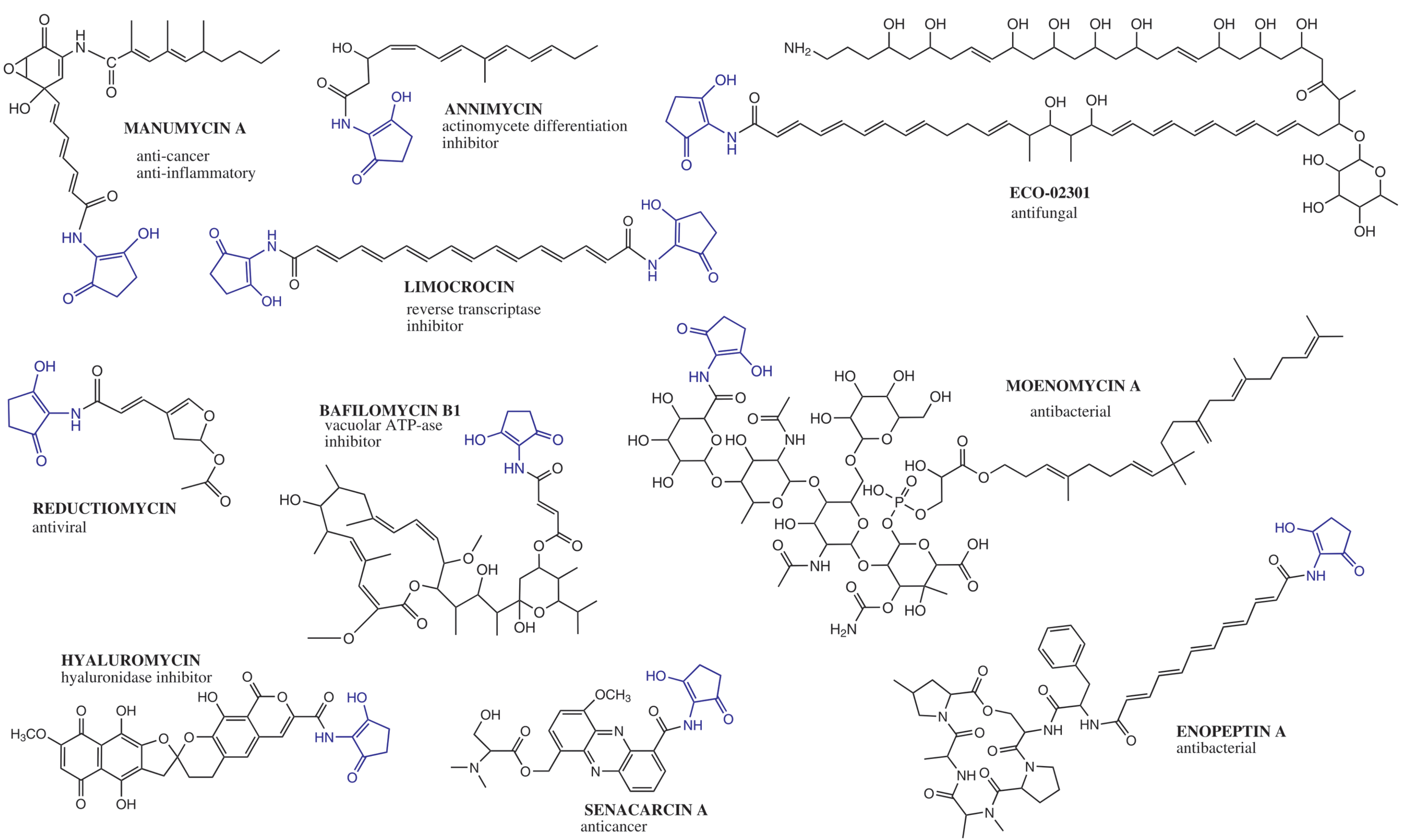

Supplementary Figure 3. BOX-PCR profiles of 234 streptomycete-like environmental isolates. Left, complete linkage clustering of the isolates based on the similarity matrix of their BOX-PCR profiles calculated using Pearson product moment coefficient. BOX-PCR groups are listed along the red line, indicating similarity threshold (set up at 70%). Right, isolate names with color-dependent assignment to the *hemA* gene phylogenetic clusters (see in Figure 4): pink – "purple" branch, dark and light green – "green" branch (moenomycins and bafilomycins, respectively), brown – "brown" branch, orange – "orange" branch, grey – unknown metabolites, blue – "blue" branch, black – strains with negative *hemA* amplification result.

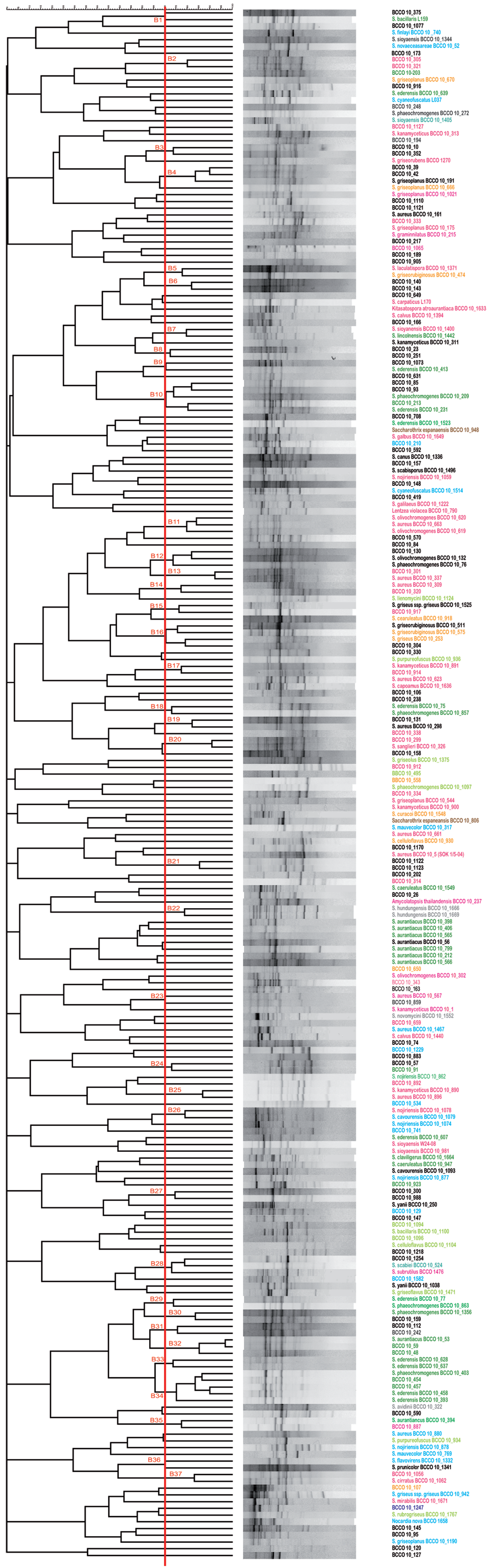

Supplementary Figure 4. Amino acid sequence alignment derived from *hemA* PCR fragments of the strains from orange (c) and brown (b) branches. Separately standing sequences from the polyketide group (highlighted yellow in Figure 4) are also included **(a)**. Conservation of the cyclizing ALAS-typical amino acid residues boxed in grey or black in case of directly activity-connected residues, corresponds to Figure 1.

```
          10        20        30        40        50        60        70        80        90        100
VMDAML RAVERFGAGSGGSRNI GGTNHVHVRLEEELAALHGKEAALLFTSGYTANDGALTVLAGAAPDSVVFSDELNHASI I DGLRHSGADKRI FRHNDT   BCCO322                      a
VLEAMKQAVDEYGAGSGGSRNI SGTNHHHVLLEKELAALHGKESALLFSSGYAANDGALTVLAGRI DDCLVFSDELNHASLI DGI RHSGAEKRI FRHNDA   BCCO1552
VLEAMKEAI DLFGAGSGGSRNI GGTNHYHVLLEKELADLHGKEEALLFSSGYTANDGSLTI LAGRLDGCVVFSDELNHASI I DGLRHSGAEKHI FRHNDV   BCCO1666, BCCO1669
VMSAMKASI DDYGAGSGGSRNI SGTNHHHVLLERELADLHGKEQALLFSSGFSANDGTLTVLASTPDDCLVLSDAKNHASI I DGLRHSGAEKQI FRHNDV   BCCO806                      b
VMSAMKSSI DDYGAGSGGSRNI SGTNHHHVLLERELAGLHGKEQALLFSSGFSANDGALTVLASTPEDCLVFSDAKNHASI I DGLRHSGAEKRI FRHNDV   BCCO948
VMAAMKGAI DDHGAGSGGSRNI SGTNHHHVVLERELAELHGKEQALLFSSGFSANDGALTVI ASTLDQCLVFSDAKNHASI I DGLRHSGAEKRI FRHNDV   S.esp DSM 44229 BN6_48530
VLAAMKDAI DRYGGGSGGSRNI AGTNPVHHVLEAELADLHGKEQGLLFGSGYSANDGALTVLAGGTENCAVFSDEKNHASI I DGLRHSGAEKHI FRHNDV   S.esp DSM 44229 BN6_50240
VLSAMKEAVERFGAGAGGSRNI AGTNHYHVALERELAELHGKEDALLFTSGYTANDGSLTVLAGRPEDCI VFSDEKNHASI I DGLRHSGVEKKI FRHNDV   A.ori HCCB10007
VLAARKKATDEAGAGSGGSRDLAGDDRHHVALEEELADLHGMESALI FPSGYSANDAALTVLAGRPPGCVVFADDLNHASMTAGI QHSGAEKHVFRHNDT   BCCO107, BCCO474             c
VLAARKKATDEAGAGSGGSRDLAGDDRHHVALEEELADLHGMESALI FPSGYSANDAALTVLAGRPPGCVVFADDLNHASMTAGI QHSGAEKHVFRHNDT   BCCO253
VLAARKKATDEGGAGSGGSRDLAGDDRHHVALEEELADLHGVESALI FPSGYSANDAALTVLAGRPPGCVVFADDLNHASMTAGI QHSGAEKHVFRHNDT   BCCO918
VLAARKKATDEAGAGSGGSRDLAGDDRHHVALEEELADLHGMESALI FPSGYSANDAALTVLAGRPPGCVVFADDLNHASMTAGI QHSGAEKHVFRHNDT   BCCO575
VLAAMKKAVDEAGAGSGGSRNI SGNNRHHVALEEELADLHGMESALI FPSGYSANDAALTVLAGRLPGCVVFSDALNHASMI AGI RHSGAEKHVFRHNDT   BCCO650
VLAAMKKAVDEAGAGSGGSRNI SGNNRHHVALEEELADLHGMESALI FPSGYSANDAALTVLAGRLPGCVVFSDALNHASMI AGI RHSGAEKHVFRHNDT   BCCO930
VLEAMKRAVDESGAGSGGSRNI SGNHRYHVELERELADLHGMESALI FPSGYAANDAALTVLAGRLPGCVVLSDELNHASMI AGI RHSGAEKQI FRHNDL   BCCO1548
VLEAMKKAVDETGAGSGGSRNI SGNNHYHVQLERELADLHGMEAALI FPSGYAANDAALTVLGSRLPGCVVLSDELNHASMI AGI RHSGAEKHI FRHNDV   BCCO558
GLAAMKRAVDEAGAGSGGSRNI SGNNRYHVELERELAELHGMEEALI FPSGYAANDAALTVLAGRLPGCVVFSDELNHASMI AGI RHSGAEKHVFRHNDT   BCCO666, BCCO670
```

```
       110       120       130       140       150
AHLEELI AAEPADRPKLI VLESVYSMSGDVAPLREVADI ARRHGATI FL   BCCO322
EHLAELLAAAPPGRPKLI VLESVYSMSGDVAPLAEI AEI ARRHGATTFL   BCCO1552
EHLEKLI AQADPTRPKLI VLESVYSMSGDLAPLAEI AEVAQRHDAMTFL   BCCO1666, BCCO1669
AHLRELLASADPARPKLI VLESVYSMSGDI APLAEI ADLADEYGAI TYL   BCCO806
AHLRELLAAADPTRPKLI VLESVYSMSGDI APLAEI ADLADEHGAI TYL   BCCO948
AHLRELLAAADPTRPKLI VLESVYSMSGDI APLAEI ADLADEYGAI TYL   S.esp DSM 44229 BN6_48530
THLRELI AAVDPARPKLI VI ESVYSMSGDLAPLAEI ADI AHEHNAVTFL   S.esp DSM 44229 BN6_50240
AHLAELLAAAPADRPKMI VFESVYSMNGDI APLAEFAALAKQYDAMTYV   A.ori HCCB10007
DHLEELLAGTEPSVPKI I ALESI YAMGSDPAPLERI AELARHHGAFAYL   BCCO107, BCCO474
DHLEELLAGTEPSVPKI I ALESI YAMGSDPAPLERI AELARHHGAFAYL   BCCO253
DHLEELLAGTEPSVPKI I ALESI YAMGSDPAPLERI AELARHHGAFAYL   BCCO918
DHLEELLAGTEPSVPKI I ALESI YAMGSDPAPLERI AELARHHGAFTYL   BCCO575
DHLEELLAGTDPSLPKI I ALESI YSMESDMAPLERI AELAAQHGAFTYL   BCCO650
DHLEELLAGTDPSLPKI I ALESI YSMESDMAPLERI AELAAQHGAFTYL   BCCO930
DHLEELLAGTDPALPKI I ALESVYSMESDVAPLERVAELAQAYGAFTYL   BCCO1548
DHLEELLAGTDPSLPKI I ALESVYSMESDVAPLERVAELAAQYGAFTYL   BCCO558
DHLEELLAGTDPGLPKLI AFESVYSMESDVAPLERI AELAETYGAFTYL   BCCO666, BCCO670
```
